# From phenotype to biology: a multi-modal roadmap of biofluid, tissue, imaging, and digital biomarkers in Parkinson’s disease

**DOI:** 10.1186/s40035-026-00558-0

**Published:** 2026-05-28

**Authors:** Zhenwei Yu, Dongning Su, Siming Li, Shinuan Lin, Kang Ren, Tao Feng

**Affiliations:** 1 Center for Movement Disorders, Department of Neurology, Beijing Tiantan Hospital, Capital Medical University, Beijing, China; 2 Department of Pathophysiology, Beijing Neurosurgical Institute, Capital Medical University, Beijing, China; 3 GYENNO SCIENCE CO., LTD., 8F, Building B2, Creative City, Chuang Ke Road, Xili Street, Nanshan District, Shenzhen, 518000 China; 4 HUST-GYENNO CNS Intelligent Digital Medicine Technology Center, School of Artificial Intelligence and Automation, Huazhong University of Science and Technology, Wuhan, 430074 China; 5 China National Clinical Research Center for Neurological Diseases, Beijing, China

**Keywords:** Parkinson’s disease, Biomarker, α-Synuclein, Seed amplification assay, Neuroimaging, Digital biomarker, Translational medicine

## Abstract

The definition of Parkinson’s disease (PD) is undergoing a profound transformation from a “clinical syndrome” to a “biological entity”, with development of two objective biological classification systems, the NSD-ISS (Neuronal Alpha-Synuclein Disease Integrated Staging System) and the SynNeurGe framework. Multimodal diagnostic tools further improve the detection of PD. This review synthesizes advances in three key domains of PD detection. First, fluid and tissue biomarkers, particularly using α-synuclein (αSyn) seed amplification assays, allow detection of synucleinopathy in cerebrospinal fluid, blood, saliva, and skin. This supports pathological diagnosis and differential classification. Extracellular vesicles provide cell-type-specific cargo profiles, while neurofilament light chain indicates neuroaxonal injury. Second, neuroimaging captures in vivo pathology. MRI identifies nigral degeneration (nigrosome-1 loss, iron accumulation, neuromelanin depletion), MRS reveals metabolic and neurotransmitter imbalances, and αSyn positron emission tomography tracers enable direct visualization of aggregation. Third, digital biomarkers derived from wearable devices, videos, and audios quantify real-world motor and non-motor symptoms, enabling continuous, ecological monitoring. Integration of these complementary biomarker streams is essential for biological definition and stratification of PD patients, and development of targeted therapies. Standardization of assays, multicenter validations, and clear guidance on who should be tested, when testing is appropriate, and how results should inform diagnosis, stratification, or monitoring are required for the translation of these methods into clinical practice in PD.

## Introduction

Traditionally, the diagnosis of Parkinson’s disease (PD) relies on the identification of motor features including bradykinesia, rigidity, and resting tremor [1]. However, the conceptualization of PD is now shifting toward a biological definition, driven by the realization that the clinical phenomenology is not sufficient for the definition of molecular pathology of PD [2–4]. To understand the significance of this shift, it is essential to trace the historical evolution of the disease’s definition.

In 1817, James Parkinson published his seminal paper “An Essay on the Shaking Palsy”, providing the first comprehensive description of the condition he termed paralysis agitans [5]. Decades later, the French neurologist Jean-Martin Charcot refined this clinical picture at the Salpêtrière Hospital. The understanding of PD pathology took another quantum leap in 2003 when Heiko Braak and colleagues proposed a staging system based on the predictable topographic spread of Lewy body pathology (misfolded α-synuclein [αSyn]) [6]. Despite these historical advances, the reliance on clinical motor symptoms for diagnosis has resulted in a critical “disconnect” between pathophysiology and clinical presentation [2]. By the time a clinical diagnosis is established, 60%–80% of dopaminergic neurons in the substantia nigra pars compacta (SNc) have already been lost, rendering neuroprotective interventions largely ineffective [7, 8]. Furthermore, PD is increasingly recognized not as a single cohesive disorder, but as a highly heterogeneous cluster of disorders with varying genetic, molecular, and pathological drivers [2–4, 9]. The heterogeneity of patients with different underlying biologies (e.g., *LRRK2* mutations, *GBA* variants, or idiopathic forms) in the same cohorts is now recognized as an important driver for the failure of disease-modifying clinical trials to date [2, 9, 10].

This historical trajectory sets the stage for the 2024 paradigm shift: the transition to a biological definition. To enable precision medicine, the field is moving away from the subjective assessment of symptoms toward the objective biological classification systems like the Neuronal Alpha-Synuclein Disease Integrated Staging System (NSD-ISS) and Synucleinopathy-Neurodegeneration-Genetics (SynNeurGe) [2, 3]. These frameworks define the disease by its biology (the presence of pathogenic αSyn and neurodegeneration) rather than its late-stage clinical consequences. This transition depends on the validation of robust biomarkers capable of detecting specific molecular pathologies, tracking disease progression, and distinguishing PD from atypical parkinsonism with high specificity [9, 11]. In this review, we synthesize the multimodal group of fluid- and tissue-based biomarkers, as well as imaging and digital biomarkers required to finally realize the promise of precision neurology.

## Conceptual framework restructuring: the dialectic and integration of two major systems

NSD-ISS and SynNeurGe, two frameworks proposed in 2024, are pioneering biological classification systems that redefine PD as a biological entity [2, 3]. They represent two distinct, yet interconnected, philosophical perspectives on the nature of PD, moving the field from a purely clinical syndromic model toward a profound transformation based on definitions.

### Conceptual divergence between NSD-ISS and SynNeurGe: disease redefinition vs biological classification

The NSD-ISS framework, proposed by Simuni et al., is centered on a radical redefinition [3]. It advocates that the traditional clinical term “Parkinson’s disease” should be replaced by a biomarker-defined precise entity—”Neuronal Alpha-Synuclein Disease (NSD)”[3]. Its diagnosis follows a strict, exclusive formula: NSD = S^+^ (αSyn pathology) + D^+^ (dopaminergic dysfunction). This system constructs a linear disease progression model from Stage 0 (genetic risk) to Stage 6 (severe disability), emphasizing the continuous evolution of biological abnormalities. However, its exclusivity is a primary point of controversy: it excludes approximately 10%–15% of patients with clinically typical PD (e.g., carriers of *LRRK2* or *Parkin* mutations, who often test negative on αSyn seed amplification assays [αSyn-SAAs]) from an NSD diagnosis, sparking criticism that its definition is overly narrow [9, 10]. Furthermore, some scholars point to a potential risk of “circular reasoning”—the assumption that biomarker positivity equates to clinical disease, overlooking the possibility that such pathology may remain asymptomatic for a lifetime [2, 10].

In contrast, the SynNeurGe system, proposed by Höglinger et al., embodies a philosophy more inclined toward inclusive description and classification [4]. It does not seek to replace clinical diagnosis but rather provides a three-dimensional matrix (S-synuclein pathology/G-genetics/N-neurodegeneration) to characterize the unique biological profile of each individual. This is a framework that embraces heterogeneity. For example, a patient who tests negative for αSyn pathology but has a specific genetic mutation and typical neurodegeneration could be classified as S^−^ G^+^ N^+^. Thus, SynNeurGe functions more as a “biological map” for research, acknowledging multiple pathways to PD rather than forcing all patients into a single linear trajectory. Key conceptual differences between the NSD-ISS and SynNeurGe frameworks, including their aims, biological criteria, and intended clinical applications, are summarized in Table 1.

**Table 1:** Comparison of the NSD-ISS and SynNeurGe frameworks for Parkinson’s disease

Feature	NSD-ISS [3]	SynNeurGe [4]
Definition	Disease definition & biological staging	Disease classification system
Core biomarkers	S+ (Synucleinopathy) & D+ (Dopaminergic deficit)	S (Synuclein)–N (Neurodegeneration)–G (Genetic)
Inclusion criteria	Restricted to individuals with Lewy body pathology (excluding SAA^–^ cases)	Encompasses all subtypes with clinical/biological features of PD
Primary use	Design of disease-modifying therapy (DMT) clinical trials	Exploring disease heterogeneity and pathological mechanisms in research
Role of clinical features	Used to assign clinical stages after biological definition	Used to describe the clinical phenotype alongside S, N, and G biological features
Key updates (2024)	Introduction of specific anchor points (MDS-UPDRS, MoCA) for staging	No major updates reported

Abbreviations: *NSD-ISS* Neural Synuclein Disease Integrated Staging System, *SynNeurGe* Biological classification based on Genetics, Synuclein, and Neurodegeneration, *PD* Parkinson’s disease, *MDS-UPDRS* Movement Disorder Society Unified Parkinson’s Disease Rating Scale, *MoCA* Montreal Cognitive Assessment

### Key evolution: clinical anchors in NSD-ISS

A core weakness of the initial version of NSD-ISS was its disconnect from clinical manifestations—it clarified “biological disease” (Stage 2) but did not clearly define the transition to “clinical disease”[3]. Leveraging data from several large perspective cohorts, the updated version introduced clinical anchors based on motor and cognitive functional impairment, making the staging system clinically operational. For motor function anchor, specific score thresholds on the MDS-UPDRS (e.g., Part II for daily activities or Part III for motor examination) were established as the core criteria for progression from Stage 2 (prodromal) to Stage 3 (functional impairment, marking clinical onset). For cognitive function anchor, corresponding thresholds on the MoCA score were concurrently introduced to define relevant cognitive decline [12].

This evolution is important. It transforms NSD-ISS from a theoretical biological framework into a practical tool applicable for clinical trial design, patient stratification, and targeted intervention. With this tool, researchers can now precisely identify the patient population at a critical stage of being “biologically positive with newly quantifiable functional impairment”. This is essential for testing the efficacy of disease-modifying therapies within this time window, marking a pivotal step towards the practical implementation of precision medicine in PD.

Implementation of such frameworks requires a multimodal group of biomarkers, including body fluid- and tissue-based biomarkers, neuroimaging biomarkers, and digital biomarkers. Biomarkers present in body fluids and tissues are employed to detect S^+^ lesions (αSyn pathology); imaging biomarkers detect dopaminergic dysfunction or neurodegeneration of the nervous system; and digital biomarkers detect clinical features such as motor function.

## Body fluid- and tissue-based biomarkers

Clinical diagnosis of PD remains imperfect in early disease and in atypical presentations, and the rate of misclassification may exceed clinically acceptable thresholds in real-world settings. Biomarkers are therefore needed to (1) detect disease-defining pathology, (2) differentiate PD from atypical parkinsonism, and (3) track disease progression and treatment response. Two recent position papers on NSD/NSD-ISS and SynNeurGe, respectively, formalize a biology-first view of PD, in which αSyn pathology (S), neurodegeneration (N), and genetic contribution (G) are integrated to define and stage disease for research and, increasingly, for clinical translation [3, 4]. In the following, we summarize body fluid- and tissue-based biomarkers emphasizing four translationally mature areas: (1) biopsy and αSyn-SAA, (2) extracellular vesicle (EV) biomarkers, (3) atypical parkinsonism differential diagnosis, and (4) co-pathology biomarkers (amyloid/tau). We additionally highlight emerging peripheral tissue SAAs and the key requirements for reliable clinical use, including sampling, assay standardization, and multicenter validation [13–16].

### Biopsy and αSyn-SAAs

**Fig. 1:**
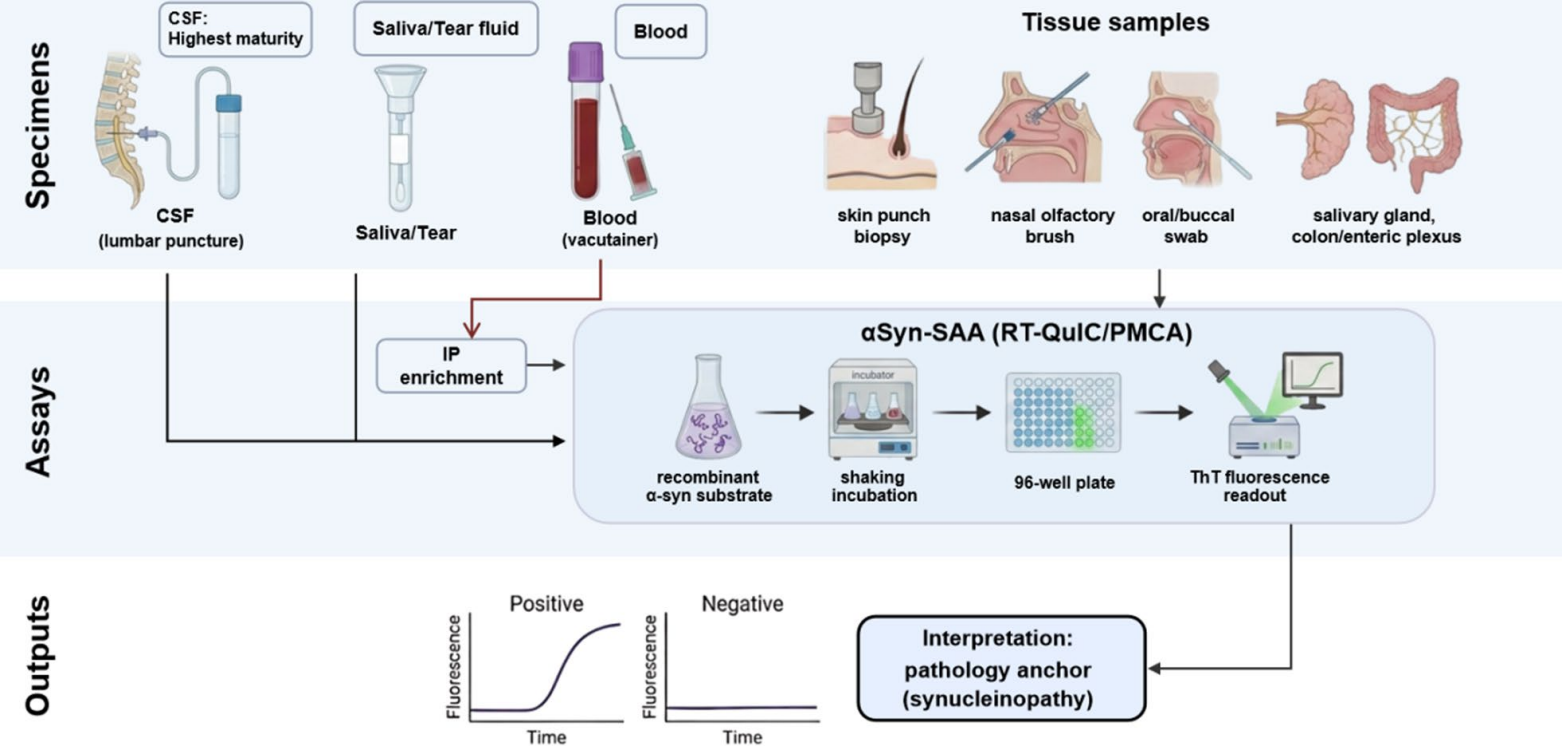
α-Synuclein (αSyn) pathology detection across biofluids and tissues. Schematic overview of αSyn pathology detection across accessible matrices. Biofluid specimens (CSF, blood, saliva, tear fluid) and peripheral tissues (skin, olfactory mucosa, oral mucosa, salivary gland, gastrointestinal/enteric plexus) feed into αSyn seed amplification assays (αSyn-SAA; RT-QuIC/PMCA formats) and/or immunodetection approaches (e.g., phosphorylated/aggregated αSyn in tissue). Outputs are shown as representative positive versus negative kinetic curves and a pathology-anchored interpretation supporting synucleinopathy biology, suitable for diagnosis support, prodromal enrichment, and trial stratification

Total αSyn immunoassays in CSF and blood have shown limited diagnostic value when used alone, due to the overlap between groups, blood contamination, and dependence on the αSyn species measured. Accordingly, contemporary strategies focus on either measuring disease-relevant conformers (oligomeric/phosphorylated species) or amplifying pathological seeds using SAA technologies [13] (Fig. 1).

#### Biofluid αSyn-SAAs

αSyn-SAAs, including real-time quaking induced conversion (RT-QuIC) and protein misfolding cyclic amplification (PMCA), detect misfolded αSyn by utilizing the intrinsic self-replicative nature of misfolded αSyn aggregates (seeds). Trace amounts of seeding-competent misfolded αSyn in a biospecimen can catalyze the conversion of recombinant αSyn monomers into amyloid fibrils, tracked by real-time fluorescence kinetics. The assay performance is highly dependent on protocols (substrate sequence/preparation, buffer composition, cofactors, shaking/incubation cycles, and positivity thresholds), which can influence not only the sensitivity and specificity but also strain selectivity (e.g., preferential amplification of Lewy-type vs MSA-type seeds) and cross-laboratory reproducibility [13, 17]. Accordingly, interpretation should be indication- and matrix-specific. Harmonization of pre-analytics, quality control, and standardized kinetic reporting are increasingly viewed as prerequisites for broad clinical use [14, 15].

Recognizing the impact of protocol variations, the field is now shifting its focus toward standardizing αSyn-SAAs for clinical use. To facilitate the transition from heterogeneous research protocols to clinically deployable (e.g., CLIA-grade) testing, a recent consensus outlines critical steps, including: (1) data sharing that includes aggregation kinetics data and pre-analytical raw data, (2) a consensus on which specific parameters or combination of parameters should be reported, and (3) establishing universal reference samples as standard calibrations to evaluate the assay performance across laboratories [14].

Recombinant αSyn substrate preparation (purity, monomerization state, and lot-to-lot stability), shaking/incubation procedures, and buffer composition (including pH and ionic strength) can substantially shift kinetic readouts such as lag time and ThT maxima, thereby generating inter-laboratory variability [14].

Although current αSyn-SAAs are mostly reported as binary positive/negative results, quantitative extensions are rapidly emerging. End-point dilution analysis (e.g., SD50 estimation), kinetic calibration approaches, and digital seed amplification assays (which partition the reaction into microcompartments to detect single αSyn) can improve the precision of quantitation. They may be used as pharmacodynamic biomarkers of target engagement and treatment response in disease-modifying trials [14, 18– 20]. Representative αSyn-SAA protocols and the major sources of inter-laboratory variability are summarized in Table 2.

**Table 2:** Representative αSyn-SAA protocols and limitations

Platform	Specimens	Assay features	Strengths	Limitations
Amprion αSyn-SAA (formerly SYNTap)	CSF (PPMI Gold Standard)	Substrate: Wild-type α-synuclein; Condition: Optimized shaking (evolution from original sonication); specific bead/buffer mix; Readout: Fluorescence kinetics (Tmax, AUC) Source: Commercial/Centralized (Amprion)	Highest sensitivity: Validated in PPMI (*n* > 1000); excellent detection of “S+ ” in prodromal and early PD; Standardization: Highly standardized SOPs; minimal inter-batch variability due to centralization; Regulatory: FDA Letter of Support; CLIA-validated	Access: Proprietary nature and centralized testing model hinder independent validation and replication by academic laboratories Strain specificity: Lower sensitivity for MSA compared to PD
RT-QuIC (Classic/Academic)	CSF (Research & Specialized Clinical Centers)	Substrate: Often K23Q mutant (faster kinetics) or Wild-type Method: Double-orbital shaking; Temp > 42 °C Readout: Lag phase, Vmax	Validation: Extensively replicated globally across academic centers; Specificity: Excellent (> 95–99%); Versatility: Protocols adaptable for strain discrimination (e.g., MSA vs PD profiles)	Variability across labs in recombinant protein preparation and shakers; Substrate bias: K23Q mutant may miss certain pathological conformers compared to wild-type
Tissue SAA	Skin biopsy, mucosa peripheral	Pre-processing: Homogenization (bead beating) & digestion essential to release seeds Substrate: Typically wild-type	Minimally invasive compared to lumbar puncture; Subtyping: Distinguishes Body-first (high sensitivity) vs Brain-first PD; Accuracy: High concordance with CSF in recent 2025 studies	Interference: Tissue lipids/blood can inhibit reaction; Sampling: Site selection is critical to avoid false negatives in Brain-first cases Inconsistent tissue homogenization methods compromise inter-laboratory reproducibility
Blood-based SAA (IP-RT-QuIC, EV-SAA)	Serum/plasma	Enrichment: Critical upstream step (Immunoprecipitation or EV capture) to concentrate seeds and remove albumin Amplification: Extended cycles often required	Scalability: Potentially suitable for large cohort studies and biomarker-enriched trial recruitment after analytical validation; Patient burden: Lower procedural burden than CSF collection; Throughput: Ideal for large pharmaceutical trials	High technical barrier (extremely low seed vs high background) Reproducibility: Enrichment efficiency (e.g., antibody clones, EV methods) varies widely; Still largely Research Use Only (RUO)
Quantitative / Digital SAA (Emerging)	CSF, Biofluids Pharmacodynamics (Target Engagement)	Method: Endpoint dilution (SD50) or Digital partitioning (nanowells/droplets); Readout: Absolute quantification of seeding units	Converts binary +/- into quantitative data; Monitoring: can potentially track therapy-induced reduction in seed load	Cost/Complexity: Requires specialized microfluidic equipment; Validation: Longitudinal correlation with clinical decline remains to be established

##### CSF αSyn-SAAs

CSF is currently the most widely used biological specimen for αSyn-SAAs [21, 22], providing an analytically favorable background and proximity to central nervous system (CNS) pathology. In a meta-analysis, CSF αSyn-SAA robustly detects seeding activity in Lewy body synucleinopathies and shows high diagnostic accuracy versus controls and non-synucleinopathies. This supports its use for diagnostic confirmation, biologically anchored cohort stratification, and clinical-trial enrichment [16, 23]. Importantly, αSyn-SAA should be conceptualized as a test for underlying αSyn pathology rather than a one-to-one surrogate for any single clinical syndrome; therefore, discordant clinical–biological profiles (e.g., parkinsonism with negative CSF αSyn-SAA, or atypical phenotypes with positive CSF αSyn-SAA) should prompt careful re-phenotyping and consideration of mixed or evolving pathology [14, 15]. A recurring observation is the substantially lower sensitivity for MSA in several CSF SAA datasets, which is consistent with the presence of distinct αSyn strains in MSA CSF and/or the reaction conditions that preferentially amplify Lewy-type seeds [16, 24, 25]. Protocol innovations in substrate variants, reaction conditions, or tailored kinetic cutoffs may improve MSA detection, but studies for multi-center replication and inter-laboratory comparability are needed before routine clinical use [17]. For clinical translation, recent reviews emphasize the importance of standardized sample handling (collection tubes, time-to-processing, centrifugation, storage temperature, and freeze–thaw limits), transparent reporting of kinetic metrics (lag time/threshold crossing, maximum fluorescence, area-under-curve), and use of well-characterized positive/negative controls and replicate wells as minimum reporting standards [13–15].

In August 2024, the U.S. FDA (Center for Drug Evaluation and Research) issued a Drug Development Tool Letter of Support (DDT-BMQ-000157) to the Critical Path for Parkinson’s (CPP) consortium, encouraging further studies and use of αSyn-SAAs in human CSF to improve the efficiency of early-intervention clinical trials for neurodegenerative diseases defined by a shared synuclein biology [26]. Notably, the letter positioned binary αSyn-SAA positivity as a biomarker of susceptibility/risk enrichment that can be used to select participants who are biologically positive for synuclein pathology, independent of the clinical syndrome. This explicitly aligns with the biology-first staging systems (e.g., NSD-ISS). This regulatory signal reframed αSyn-SAAs from an investigational research assay to a practical trial-enabling tool: by enriching for synuclein-positive participants, studies can reduce pathological heterogeneity (including clinically similar but synuclein-negative presentations), thereby increasing power to detect disease-modifying effects.

In parallel, αSyn-SAAs are already offered clinically as CAP/CLIA laboratory-developed testing (e.g., Amprion SAAmplify-αSYN) [27, 28], and are increasingly embedded in therapeutic development programs as an exploratory or enrichment tool. This highlights the urgency of cross-platform reference standards and transparent reporting to ensure that “positive/negative” calls are comparable across settings.

##### Blood αSyn-SAAs

Blood-based αSyn-SAAs aim to extend seeding readouts beyond lumbar puncture but face major analytical barriers, including extremely low seed burden, high background protein/lipid content, and potential inhibitors of fibrillization [29]. To address these constraints, most reported blood workflows incorporate an upstream seed enrichment step (e.g., immunoprecipitation of αSyn species, capture of neuron-enriched EV fractions, or other affinity-based concentration steps) followed by RT-QuIC readout, with performance dependent on both enrichment efficiency and downstream reaction conditions [30]. Pre-analytical variables that are relatively modest for CSF can become dominant, including plasma versus serum selection, hemolysis, platelet activation, tube additives, processing delays, storage duration, and repeated freeze–thaw cycles; these factors can alter αSyn abundance, introduce interfering species, or generate false-positive/false-negative kinetics [14, 15]. Early studies combining enrichment with RT-QuIC reported encouraging discrimination of synucleinopathies from controls and suggested feasibility for scalable screening and trial enrichment. However, the heterogeneity in protocols and outcome definitions currently limits cross-study comparability and clinical generalizability [30]. Accordingly, head-to-head protocol comparisons, specimen-specific quality control processes, external validation in multi-center cohorts, and harmonization of kinetics reporting (including prespecified thresholds and handling of borderline curves) are needed in priority [14, 15].

##### Saliva αSyn-SAAs

Saliva represents an attractive, non-invasive specimen for αSyn-SAAs, with potential utility for population-scale sampling and longitudinal monitoring [31, 32]. However, its complex and variable composition may introduce substantial technical variability. Salivary flow, the presence of mucins and proteases, microbial content, and intermittent blood contamination can all modulate seed recovery and reaction kinetics. Therefore, standardized collection (collection time of the day, fasting status, stimulation, oral hygiene), immediate stabilization, and defined preprocessing (clarification, concentration, and/or seed enrichment) are critical for the reproducibility [14, 15]. Compared with CSF, saliva is expected to contain lower concentrations of seeding-active species, so its performance relies heavily on the enrichment strategies and stringent criteria for defining positivity from kinetic curves. In this context, saliva-based assays conceptually bridge biofluid and tissue approaches. Biologically, the signal may reflect peripheral autonomic and cranial innervation, as well as oral–nasal mucosal involvement. Methodologically, these assays utilize workflows adapted from tissue/mucosal SAAs (e.g., careful avoidance of surface contamination and the use of matrix-matched controls). Although published cohorts report promising diagnostic signals, broader validation remains limited and protocol heterogeneity is substantial; thus, saliva αSyn-SAAs should currently be considered investigational. Multi-center standardization, blinded replication, and harmonization of kinetics reporting and quality control metrics are required before routine clinical use [14, 15].

##### Tear fluid αSyn-SAAs

Tear fluid is another emerging, non-invasive specimen of interest. Early studies have reported altered levels of total and oligomeric αSyn in PD [33, 34], and more recent work suggests that tear fluid αSyn-SAA may detect seeding activity in a subset of PD cases [35]. However, evidence remains limited and further validation is required before clinical application.

#### Tissue-based biopsy and αSyn-SAAs

Peripheral tissues provide an additional clinically accessible peripheral window into αSyn pathology and may complement biofluid assays in selected diagnostic or research settings. Peripheral tissues can be used for (1) immunohistochemical/immunofluorescent detection of oligomeric, phosphorylated, or aggregated αSyn and (2) αSyn-SAAs (tissue homogenates or swabs). For the former, the performance depends on site selection and sampling depth, fixation, sectioning, staining, reader training, and blinding, while for the latter the αSyn-SAA/RT-QuIC readouts depend on pre-analytical handling and seed-enrichment steps. For both, standardized operating procedures and blinded inter-laboratory validation remain prerequisites for reproducibility of sensitivity/specificity and for clinical translation [21, 22].

##### Skin biopsy and SAAs

Skin biopsy is currently the most clinically mature tissue-based method for detecting pathological αSyn. The dominant approach uses immunohistochemistry or immunofluorescence to detect phosphorylated αSyn (pS129 αSyn) within cutaneous autonomic nerve fibers. The detection of pS129 αSyn is interpreted with reference to a pan-axonal marker (e.g., PGP9.5) to confirm localization to nerves. A multi-site sampling strategy (commonly distal leg, proximal thigh, and cervical/paravertebral regions) is often used to address the patchy distribution of pathology and increase sensitivity. A large cross-sectional study reported high detection rates of cutaneous phosphorylated αSyn across clinically defined synucleinopathies (PD, MSA, dementia with Lewy bodies [DLB], and pure autonomic failure) with a low positivity rate in controls, supporting strong case–control discrimination [36]. More recently, a blinded, multicenter prospective study enrolling patients with very early parkinsonism (< 18 months) reported that the baseline skin biopsy for intraneural p-αSyn in those with a final diagnosis of PD after 18–46 months of follow-up, was often positive before clinical diagnostic criteria were met, and showed high longitudinal consistency on repeat biopsies [37]. While the performance in real-world “all-comers” movement clinics may be lower than in enriched cohorts, accumulating data suggest that skin pS129 αSyn can be a practical tool for increasing the diagnostic confidence in uncertain parkinsonism, particularly when the key question is whether a synucleinopathy is present [36, 37]. Skin pS129 αSyn is expected to be positive in synucleinopathies and negative in primary tauopathies (e.g., progressive supranuclear palsy [PSP]/corticobasal syndrome [CBS]) and many non-synuclein degenerative conditions, making it conceptually analogous to CSF αSyn SAA as a “synuclein anchor”. However, it per se does not distinguish PD from other synucleinopathies (notably MSA and DLB). Therefore, if the clinical problem is PD vs MSA or PD vs DLB, skin biopsy should be combined with phenotype, autonomic profile, imaging, and other biomarkers such as NfL, which tends to be higher in atypical parkinsonism than in PD.

A fast-evolving complementary direction is applying αSyn SAAs to skin and other non-CSF tissues. Early work suggests that the seeding activity can be detected in non-CSF specimens, though the performance varied by specimen type and pre-analytical handling [22]. As with the CSF assays, standardization (sampling depth, storage, homogenization, substrate, kinetic thresholds) and blinded multi-center validation are key barriers to translation. Skin biopsy requires careful standardization and consistency in biopsy site(s), depth, fixation, staining protocol, and interpretation criteria. False negatives may arise from sampling error, early disease, or methodological variability; occasional false positives have been reported in controls in some cohorts. These underscore the need for strict quality control and blinded reads. Beyond binary positivity, emerging data suggest that cutaneous αSyn pathology may carry topographic and fiber-type “signatures” that could stratify PD into putative body-first and brain-first subtypes [38]. Finally, the practical role of skin biopsy may differ across clinical contexts: it may be most useful as a “rule-in synucleinopathy” test in diagnostically uncertain cases, rather than as a population-screening tool.

##### Gastrointestinal biopsy

Constipation is among the most common and often earliest non-motor symptoms in PD. The enteric nervous system has long been proposed as a potential initiation or amplification site for αSyn pathology in at least a subset of patients. This aligns with the “body-to-brain” model in which misfolded αSyn may appear in peripheral autonomic structures and propagate centrally along connected pathways. Therefore, gastrointestinal tissue may offer evidence of early synuclein pathology. Early work reported αSyn pathology in colonic tissues obtained years before the onset of cardinal motor symptoms, supporting the concept of a premotor enteric signal in at least some individuals [39, 40]. Studies in prodromal cohorts, including idiopathic REM sleep behavior disorder (iRBD), further suggest that the enteric synuclein pathology can precede or parallel early neurodegeneration [41].

Gastrointestinal studies have examined pS129 αSyn pathology in biopsies from the colon and upper gastrointestinal tract, as well as broader αSyn immunoreactivity patterns. However, systematic evaluations and meta-analyses emphasize that the diagnostic performance is highly sensitive to biopsy location (upper vs lower GI), tissue layer (mucosa vs submucosa/full-thickness), antibody choice (native vs phosphorylated epitopes), co-localization strategies (e.g., pan-neuronal markers), and pathology scoring criteria—factors that likely contribute to the marked inter-study heterogeneity reported to date [42–45]. Accordingly, despite strong mechanistic interest, gastrointestinal biopsy remains less mature as a diagnostic test than skin biopsy.

Key obstacles include (1) heterogeneous and patchy pathology; (2) variable representation of enteric nerves in routine mucosal biopsies; (3) non-specific staining if protocols are not optimized; and (4) uncertain incremental value over skin or CSF-based approaches in most clinical contexts. Indeed, several cohorts have reported limited or inconsistent discrimination between PD and controls using conventional immunostaining approaches, underscoring the need for rigorous standardization and blinded validation [46, 47].

A complementary and fast-evolving direction is applying αSyn-SAAs to gastrointestinal tissue, which may improve the specificity by detecting seeding-competent conformers rather than relying solely on immunoreactivity [48, 49]. For example, SAA/RT-QuIC-based studies have demonstrated detectable αSyn seeding activity in upper gastrointestine (e.g., duodenum or stomach) biopsies in PD, and comparative work suggests that the performance can differ substantially across gastrointestinal sites and disease stages, with some datasets showing higher yield in gastric/upper gastrointestinal samples than in lower gastrointestinal biopsies [48, 49]. In practice, gastrointestinal tissue may be best positioned as a research tool for mechanistic stratification (e.g., gut-first signatures) rather than as a first-line diagnostic procedure, as obtaining these samples typically requires a clinically indicated endoscopy.

##### Salivary gland biopsy

Salivary glands (labial minor salivary glands or submandibular gland) are innervated by autonomic fibers and have been investigated for pS129 αSyn detection. Salivary gland pathology gained attention in prodromal synucleinopathy (notably isolated REM sleep behavior disorder), supporting peripheral involvement before classic motor PD diagnosis [42, 50]. Salivary gland biopsy can provide direct tissue evidence of synuclein pathology, but it is more invasive than skin sampling, and diagnostic yields have been variable across studies due to the differences in gland type, technique, tissue quality, and pathology criteria. A recent systematic review/meta-analysis focusing on methods of salivary gland biopsy highlights both promise and substantial methodological heterogeneity [50]. Clinically, salivary gland biopsy is therefore less commonly pursued than skin biopsy. It is best reserved for selected scenarios such as when other tests are inconclusive and the specific salivary gland biopsy expertise is accessible, or in research settings that employ strictly standardized procedures for tissue processing and pathological evaluation [50].

##### Olfactory mucosa and oral mucosa

Hyposmia is one of the most prevalent and earliest non-motor symptoms in PD, and olfactory structures are among the earliest affected in Braak-like staging concepts. This has motivated sampling of the olfactory mucosa using nasal brushings/swabs and applying αSyn SAA to detect seeding activity. Experimental and translational studies showed that olfactory mucosa samples can seed αSyn aggregation in RT-QuIC and may even carry strain-like information that helps differentiate PD from MSA in some settings (based on kinetic/biochemical properties of the amplified product), although broader replication is needed [17, 51]. Even more accessible than olfactory mucosa, the oral mucosa has recently emerged as a feasible substrate for αSyn SAA. A recent study evaluated oral mucosa αSyn SAA in synucleinopathies and isolated REM sleep behavior disorder, showing that oral mucosal sampling could serve as a minimally invasive tool for detecting synucleinopathy biology [52]. Mucosal SAAs are appealing because they may offer an “SAA-like” biological anchor without lumbar puncture. However, performance can be sensitive to the sampling technique, contamination, specimen handling, and assay thresholds, and the evidence base is still smaller than CSF SAA.

### EVs: from separation to single-EV precision analysis

EVs, including exosomes and microvesicles, are lipid bilayer-enclosed particles that carry proteins, lipids, and nucleic acids and protect their cargo from extracellular degradation. As EVs are released by both CNS and peripheral cells and are detectable in CSF, blood, saliva, and urine, they offer a biologically plausible route for sampling PD-related molecular signatures from accessible fluids. Critically, EVs in the blood are from a mixture of sources (e.g., brain, endothelium, immune cells, platelets, and other tissues); thus, EV studies on CNS-derived EVs must explicitly address the origin, contamination, and normalization [53–58].

**Fig. 2:**
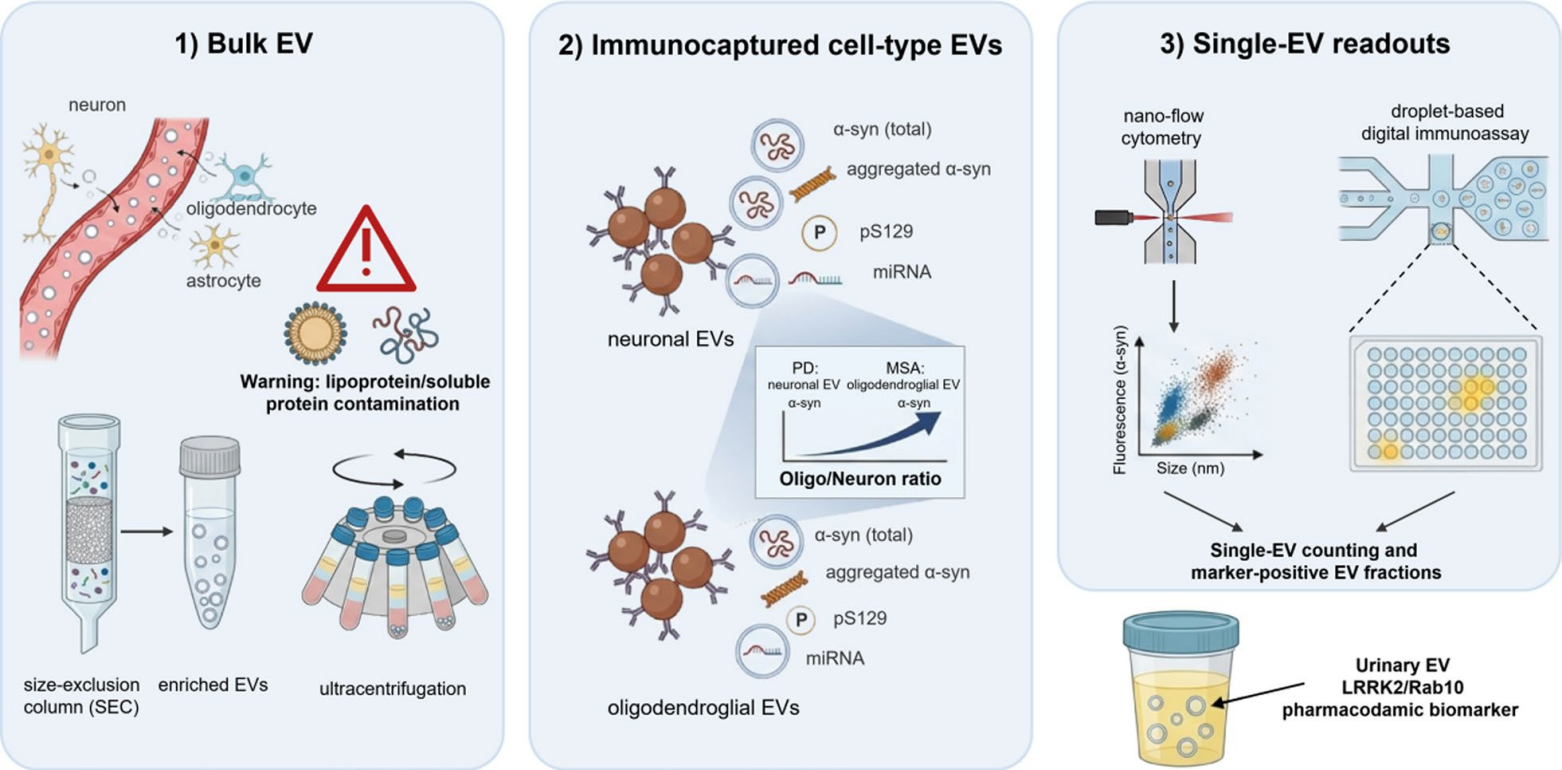
Conceptual framework for extracellular vesicle (EV) biomarkers in Parkinsonian disorders. EVs released from diverse cellular sources (neurons, oligodendrocytes, astrocytes, immune cells, platelets) carry proteins and nucleic acids, including α-synuclein species (total, aggregated, and phosphorylated forms) and regulatory RNAs. The figure shows three analytical strategies with increasing biological specificity: (1) bulk EV isolation (e.g., ultracentrifugation or size-exclusion chromatography) with potential confounding lipoprotein/soluble protein contaminants; (2) immunocapture of putative CNS-relevant EV subfraction, enabling cell-type-informed readouts; and (3) single-EV technologies (nano-flow cytometry, droplet digital immunoassays) that quantify marker-positive EV fractions and per-EV cargo. The inset highlights a differential diagnostic concept whereby neuronal EV α-synuclein signals may be relatively enriched in PD, whereas oligodendroglial EV α-synuclein signals may be relatively enriched in MSA, motivating cell-type ratios as supportive classifiers

Biomarker studies of EVs in PD typically follow three strategies: (1) measuring cargo in bulk EV preparations from plasma/serum or CSF; (2) enriching for putative CNS-originating EVs using neuronal- or oligodendroglial markers to improve the signal-to-noise ratio; and (3) deploying ultrasensitive single-EV readouts (nano-flow cytometry, digital immunoassays, microfluidic platforms) to detect low-abundance pathogenic species in defined EV subpopulations [53–56] (Fig. 2).

The performance of all three strategies is highly sensitive to pre-analytics and isolation. EV subsets isolated by differential ultracentrifugation, size-exclusion chromatography (SEC), polymer precipitation, and microfluidic/immunoaffinity capture overlap but are not identical. They also suffer different degrees of contamination by lipoproteins and soluble proteins, an issue that is particularly important for αSyn quantification in plasma. The MISEV2018 guidelines and the EV-TRACK reporting framework highlight minimal requirements for EV characterization (particle sizing/counting, EV marker confirmation, and assessment of major contaminants) and standardized reporting of sample handling (processing delay, platelet depletion, hemolysis, storage, and freeze–thaw cycles) [59, 60].

“CNS-enriched” workflows most commonly use immunocapture against surface proteins such as L1CAM, NCAM, or other cell-type–associated antigens to isolate EVs directly from plasma/serum. While this approach has generated promising diagnostic, prodromal, and differential diagnostic signals, marker specificity remains debated. This is because that some neuronal antigens may also exist in blood in a soluble or a non–EV-associated form and that the capture efficiency can vary with the antibody clone, the epitope accessibility, and the EV subpopulation targeted. Recent reviews and opinion pieces therefore recommended treating these fractions as “putative neuronally enriched EVs”, requiring orthogonal validation (e.g., EV marker colocalization, proteomic enrichment, or single-EV imaging) and cautious clinical interpretation [56, 61].

A field-defining controversy emerged in 2021 when Norman and colleagues reported that L1CAM in human plasma and CSF is predominantly present as a soluble protein rather than being associated with EVs, challenging the assumption that L1CAM immunocapture reliably isolates bona fide neuron-derived EVs [61]. This finding raised the possibility that some earlier “neuron-derived extracellular vesicle (NDEV)” biomarker signals may reflect co-isolation of soluble proteins or other non-EV particles, causing a reproducibility crisis for bulk immunocapture workflows.

To address this problem, methodology is switching toward orthogonal validation (e.g., detergent sensitivity, protease protection, density gradients, and single-particle imaging) under the community guidelines such as MISEV and EV-TRACK. More importantly, single-EV analytics can be used to verify membrane association and the true co-localization of surface markers with pathogenic cargo on individual vesicles [59, 60].

αSyn is the most extensively studied EV cargo. Meta-analyses indicate that αSyn concentrations measured in putative CNS-enriched EVs, and in some studies in total EV preparations, differ between PD and controls; however, the effect size and the diagnostic accuracy vary widely across cohorts and protocols. The heterogeneity is largely attributed to differences in EV isolation method, enrichment markers used, and assay calibration [53, 54, 56]. In a plasma EV study without neuronal enrichment, αSyn in plasma-derived EVs showed diagnostic potential, but its reliability is heavily compromised by pre-analytical variations and normalization methods, highlighting the need for standardized analytical pipelines [53, 56].

Multiple groups have moved beyond “total αSyn” to pathogenic species, including oligomeric/aggregated αSyn and phosphorylated αSyn (pS129), which may better reflect conformational abnormality or disease-relevant states. Using antibody-based nano-flow cytometry, a recent study reported that the plasma levels of neuron-derived EVs positive for total αSyn, aggregated αSyn, and pS129-αSyn were increased in iRBD compared with controls, suggesting potential utility for prodromal identification and early clinical diagnosis [62].

Emerging single-EV technologies aim to reduce matrix effects and to localize biomarkers to the EV surface versus the lumen. A droplet-based microfluidic digital immunoassay identified membrane-associated αSyn on L1CAM-positive EVs immunocaptured from human serum (supported by super-resolution microscopy) and reported strong discrimination for prodromal iRBD (area under the ROC curve (AUC) ≈ 0.93) and clinically diagnosed PD (AUC ≈ 0.95) [63]. Such platforms, together with nano-flow cytometry, provide a mechanistic handle to reconcile discrepant results from bulk EV assays by quantifying defined EV subpopulations and explicitly reporting EV counts, marker positivity, and cargo metrics in individual EVs [55].

EV-based αSyn measurements have also been explored for differential diagnosis across synucleinopathies. In a multi-cohort study, αSyn measured in blood exosomes immunoprecipitated with neuronal and oligodendroglial markers distinguished PD from MSA. Notably, the ratio between αSyn concentrations in putative oligodendroglial exosomes and those in putative neuronal exosomes improved separation of PD from MSA (AUC = 0.902), when used in combination with αSyn concentration in exosomes and the total exosome concentration [64]. These results align with the cell-type-specific pathology (Lewy neuronal pathology in PD versus glial cytoplasmic inclusions in MSA) and conceptually bridge EV biomarkers with the αSyn strain/cellular-context differences [56, 64].

Beyond αSyn, EVs contain pathway biomarkers related to etiology, subtype, and therapy monitoring. Urinary EVs/exosomes have been used to quantify LRRK2 abundance and phosphorylation (including pS1292-LRRK2) and phosphorylation of Rab substrates, offering both disease-related signals and pharmacodynamic readouts for LRRK2-targeting trials [65, 66]. EV proteomic and miRNA panels have also been proposed for PD diagnosis and progression. EV pathology cargoes including amyloid-β (Aβ) and tau species may inform cognitive risk; however, the clinical utility of these multiplexed panels remains unverified due to a lack of harmonized pre-analytical protocols and blinded, multi-center validation [54–56, 67, 68].

Overall, EV biomarkers represent a rapidly evolving “liquid biopsy” layer that contains cell-type-linked pathology and signaling. Their clinical translation requires (1) transparent and standardized EV workflows, (2) orthogonal confirmation of EV identity and biomarker localization, (3) pre-registered multi-site validation with blinded analyses, and (4) integration with complementary biomarker domains (αSyn SAAs, NfL, and co-pathology markers).

### Atypical parkinsonism differential diagnosis

#### NfL and related injury markers for differential diagnosis and prognosis

NfL is a non-specific marker of neuroaxonal injury that on average shows higher levels in atypical parkinsonian disorders (APS) such as MSA, PSP, CBS/CBD than in idiopathic PD at the group level. CSF and blood studies showed that the discriminatory performance is generally stronger for PD versus MSA/PSP [69, 70], but the effect sizes vary with age, assay platform, disease duration, and case-mix. Contemporary work therefore emphasizes age-adjusted interpretation and cross-assay harmonization (including head-to-head comparisons of commercial immunoassays) for both CSF and plasma/serum NfL [71, 72, 69, 70, 73, 74].

Platform selection is increasingly practical for clinical laboratories. In a head‑to‑head comparison of major commercial assays (including Simoa, Ella, and Lumipulse) using both CSF and plasma, the diagnostic accuracy remained high in selected research cohorts, despite systematic differences in absolute concentrations [70]. However, these data should not be over-interpreted as implying stand-alone reliability for individual diagnostic decisions. Importantly, the absence of NfL value elevation cannot exclude atypical parkinsonism, particularly earlier or less aggressive cases. NfL should be viewed as a supportive biomarker rather than a rule-in or rule-out test for atypical parkinsonism.

Beyond differential diagnosis, higher baseline NfL levels predict a more aggressive course (faster motor and cognitive decline and shorter survival) across parkinsonian syndromes [75, 76]. In biomarker algorithms, αSyn-SAA (or tissue αSyn assays) can be conceptualized as confirming synucleinopathy (“S”), whereas NfL captures neurodegeneration intensity (“N”). NfL may add supportive information for differentiating PD from rapidly progressive APS even when αSyn pathology is present, such as in MSA.

#### Pathology detection with αSyn and tau biomarkers

As NfL is non-specific, its interpretability improves when paired with biomarkers of the underlying proteinopathy. CSF αSyn-SAA provides strong evidence of Lewy-type αSyn pathology and can therefore help distinguish PD/DLB and other Lewy body synucleinopathies from primary tauopathies. However, the lower sensitivity of several RT-QuIC/PMCA protocols for MSA highlights that the assay conditions may preferentially amplify Lewy-type strains [13–16]. Recent refinements further improved the differential discrimination between PD and MSA. This analytical variability aligns with the diversity of αSyn structural strains, a phenomenon that may also underlie the clinical heterogeneity of the disease [23, 24, 25, 77]. In parallel, tissue-based assays, particularly skin immunohistochemistry for phosphorylated αSyn and skin RT-QuIC, provide minimally invasive confirmation of peripheral synuclein deposition and can differentiate synucleinopathies from non-synucleinopathies with high accuracy in multicenter cohorts [36].

Recent work has extended seed amplification approaches to tauopathies that clinically mimic PD (PSP and CBS/CBD). The 4-repeat tau (4R-tau) seeding activity has been detected in skin biopsies of PSP/CBD, and skin tau-SAA (4R-focused substrates) can differentiate tauopathies from synucleinopathies and controls, with performance influenced by biopsy site and clinical phenotype [78, 79]. In addition, quantification of total tau and 4R-tau isoforms in skin lysates separated PSP/CBD from α-synucleinopathies, supporting skin as a practical “peripheral window” for both synuclein and tau proteinopathies [80].

#### Additional biofluid biomarkers beyond NfL

Other biofluid biomarkers can further refine APS differential diagnosis and capture disease biology. Unbiased CSF proteomic screening (including proximity extension assay-based panels) has highlighted candidates such as midkine and dopa decarboxylase (DDC) that may aid in PD/atypical parkinsonism stratification, complementing injury biomarkers [81, 82]. In addition, CSF ecto-GPR37 has been proposed as a PD-associated biomarker reflecting disease-relevant biology [83]. Glial activation and inflammatory biomarkers (e.g., GFAP, YKL-40) show group-level differences between PD and APS in several studies and meta-analyses, although the overlap limits stand-alone diagnostic use and standardization remains incomplete [84, 85]. Accordingly, GFAP is best interpreted as a supportive marker of astroglial activation and/or neurodegenerative comorbidity, rather than a stand-alone discriminator between PD and atypical parkinsonism. Among synucleinopathies, EV-based strategies are being explored to separate PD from MSA by analyzing cell-type-enriched EV cargo. For example, differential distribution of αSyn between neuronal and oligodendroglial exosomes in the blood has been proposed as a sensitive biomarker for distinguishing between PD and MSA [64]. Disease-specific metabolic markers (e.g., reduced coenzyme Q10 in MSA as reported in some cohorts) and autonomic-related measures may provide supportive evidence, but currently broader validation and harmonized assays are needed [74, 86].

#### Biomarker panels and practical algorithms

**Fig. 3:**
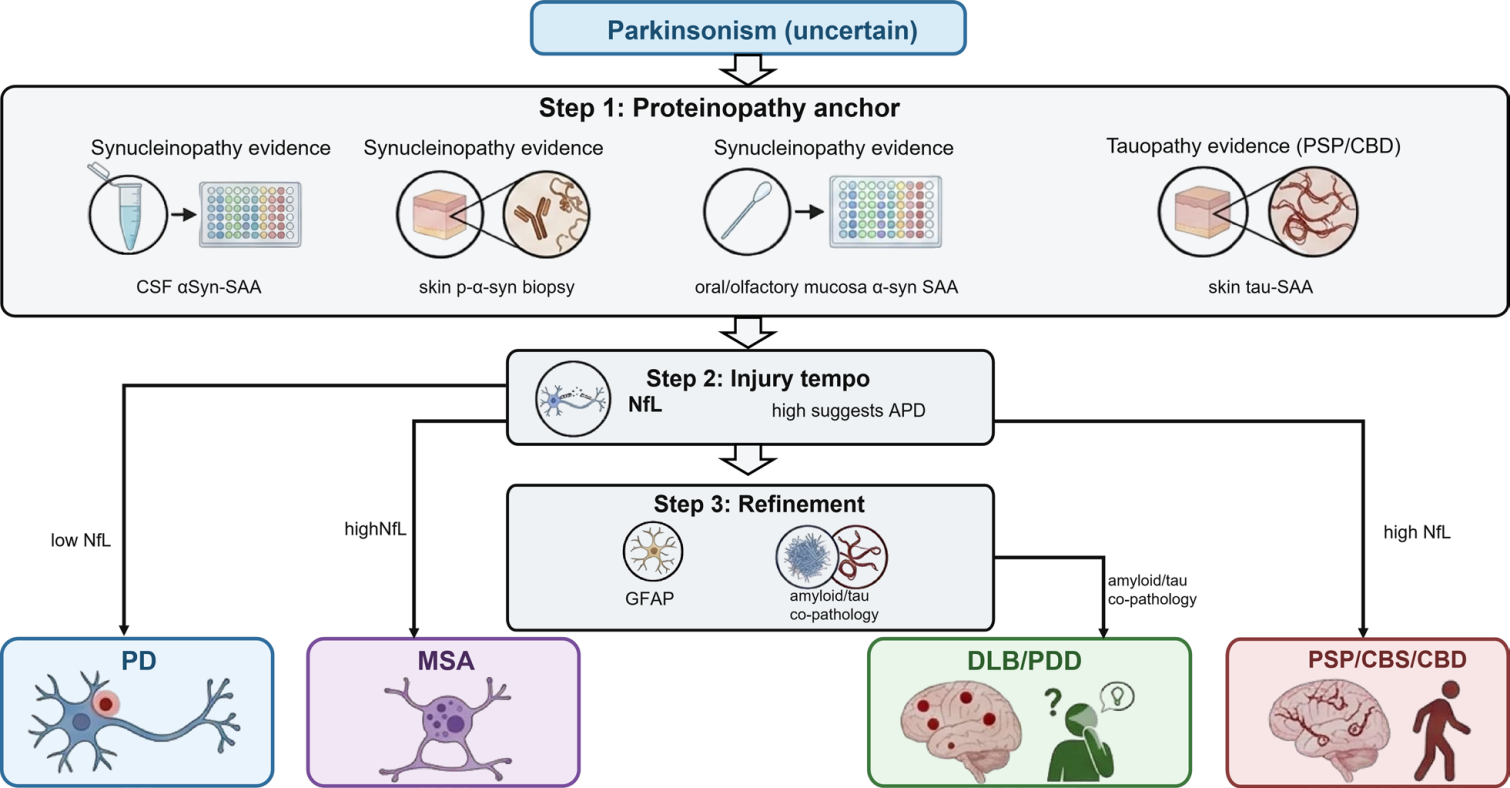
A conceptual proposition of biomarker-supported differential diagnosis of PD. Step 1 anchors synucleinopathy biology using αSyn-SAA or tissue αSyn assays. Step 2 evaluates neurodegeneration severity and atypical parkinsonism likelihood using NfL with supportive imaging where appropriate. Step 3 assesses co-pathology and supportive biology markers relevant to cognitive prognosis, multimorbidity, and trial enrichment, including amyloid/tau-related measures and, where appropriate, adjunctive glial markers such as GFAP

Clinically, biomarker panels provide the highest yield. Biomarker panels combine (1) a pathology biomarker (αSyn-SAA or tissue αSyn assay; and, where appropriate, tau-SAA), (2) an injury biomarker (NfL), and (3) supportive biomarkers (glial/inflammatory and, when cognitive symptoms are present, Alzheimer's disease [AD] co-pathology markers). Measuring biomarker panels in CNS-originating EVs can also enhance classification across parkinsonian syndromes [67]. Recent plasma panel studies combining NfL with GFAP and Aβ measures (Aβ42, Aβ40, or Aβ42/40) reported improved separation of PD from APS compared with any single marker [87]. A multimodal diagnostic workflow for parkinsonism aligning with the 2024 biological criteria frameworks is illustrated in Fig. 3. Flexible combinations (e.g., NfL with αSyn species and amyloid markers) can improve classification across parkinsonian syndromes, but prospective validation in unselected diagnostic clinics and pre-defined cut-offs are needed before routine adoption [72, 70, 87]. Within the multimarker panels, GFAP may add supportive information when combined with NfL and amyloid/tau-related measures, but its role is adjunctive rather than decisive.

### Amyloid/tau co-pathology and multimorbidity in Lewy body disorders

Cognitive outcomes in PD and DLB are strongly influenced by concomitant Alzheimer-type pathology (amyloid-beta and tau), as well as mixed proteinopathies that are common in older individuals. Recent systematic reviews conclude that the amyloid/tau co-pathology in Lewy body dementia is associated with worse outcomes, including faster cognitive decline and increased mortality [88].

Plasma and CSF biomarkers of phospho-tau and amyloid enable stratification of Lewy body disorders by AD co-pathology. A contemporary review has summarized a plasma biomarker profile in DLB, which consists of amyloid/tau, axonal injury, and neuroinflammation markers, and highlighted the value of combining co-pathology markers with αSyn-SAA to define biologically homogeneous subgroups for trials [89, 90].

## Imaging biomarkers

### Magnetic resonance imaging (MRI)

#### MRI of the substantia nigra

Substantia nigra pathology in PD is characterized by neuronal loss in the SNc. There are 5 nigrosomes of high concentrations of neuromelanin (NM) distributed non-uniformly in the SNpc. Among these, nigrosome-1 (N1) appears to be the largest and the earliest affected subregion, which is located in the dorsal medial aspect of the tail of the SNpc [91]. High-resolution susceptibility-weighted imaging (SWI) revealed that N1 is characterized by high neuronal density and relatively low iron content in healthy individuals. It is surrounded by the hypointense SNc and substantia nigra pars reticulata (SNr), which have higher iron concentrations. This configuration forms a bifurcated, swallow-tail-like appearance, known as the “swallowtail sign (STS)” [92, 93]. In PD patients, the STS disappeared due to neurodegeneration and neuronal loss in N1 followed by increased iron deposition. A meta-analysis demonstrated that the appearance of N1 on 3 T MRI has high diagnostic accuracy in differentiating PD patients from healthy adults, with an overall sensitivity and specificity of 0.94 and 0.90, respectively [94, 95]. In comparison, 7 T MRI allows a more precise characterization of the substantia nigra and visualization of its inner organization. Three-dimensional multiecho susceptibility-weighted 7 T MRI revealed a three-layered organization of the SN, which could discriminate between PD patients and healthy subjects with sensitivity and specificity of 100% and 96.2%, respectively [96]. The diagnostic specificity of the loss of STS on 3 T MRI remains controversial; therefore, 7 T MRI is recommended as the gold standard for structural biomarker research, as it can clearly resolve the microstructure of N1.

Quantitative susceptibility mapping (QSM) can also improve the accuracy of iron measurement, and has been shown to pinpoint iron accumulation specifically in the SNc. A 3 T MRI study involving 87 patients with sporadic PD demonstrated that quantitative analysis of substantia nigra pathology using QSM achieved a sensitivity of 89% and a specificity of 87% in diagnosing PD [97]. Furthermore, the substantia nigra QSM values correlated with disease duration, UPDRS Part III scores, and levodopa equivalent daily dose, indicating its potential as a reliable neuroimaging biomarker for PD [98]. 7 T MRI studies further revealed that QSM values in the substantia nigra correlated with disease duration, MDS-UPDRS total scores, and bradykinesia-rigidity sub-scores, but not with tremor or postural instability scores [99].

Neuromelanin is a dark complex pigment found in dopaminergic neurons in the SNc and the ventral tegmental area, as well as in noradrenergic neurons of the locus coeruleus. High-resolution T1-weighted imaging with fast spin-echo and magnetization transfer pulses has been used to visualize neuromelanin in the midbrain and other pigmented nuclei [100]. In PD, the substantia nigra exhibits a loss of neuromelanin-rich dopaminergic neurons. Consequently, neuromelanin-sensitive MRI shows decreased signal intensity and reduced volume of the substantia nigra in PD patients compared with healthy controls. A 2019 meta-analysis reported that neuromelanin-sensitive MRI differentiated PD patients from healthy controls with sensitivity and specificity both of 82% [101]. Longitudinal studies indicate that the neuromelanin-derived substantia nigra volume progressively declines with disease duration in PD. The neuromelanin signal changes appeared to start in the posterolateral motor areas of the substantia nigra and then progressed to more medial areas of this region [102]. The analysis of neuromelanin-sensitive MRI is transitioning from “visual rating” to “AI-assisted automated quantification”. The use of deep learning models to automatically segment SNc and calculate neuromelanin volume significantly reduces inter-observer variability and shows a strong correlation with dopamine transporter (DAT) loss [103, 104].

Diffusion tensor imaging (DTI) is an MRI technique that can indirectly evaluate the integrity of white matter tracts by measuring water diffusion and its directionality in three dimensions. A DTI-based study revealed significantly decreased fractional anisotropy (FA) in the substantia nigra and lower part of the putamen/caudate complex of PD patients, in which most of the nigrostriatal dopaminergic neurons are included. Loss of FA in this region was obvious even during the early clinical stages of PD [105]. Current evidence remains inconclusive regarding whether DTI can reflect disease progression in PD. Several longitudinal studies have demonstrated that DTI metrics can effectively predict PD progression and correlate with bradykinesia as well as cognitive function in patients. However, a 2017 meta-analysis concluded that DTI parameters show no significant association with PD disease duration [106, 107].

When evaluating structural and functional imaging, it is crucial to distinguish between established clinical tools and experimental methodologies. Standard structural MRI primarily serves to exclude secondary causes of parkinsonism (e.g., vascular lesions, tumors) and remains a routine clinical standard. Conversely, advanced structural techniques like 7T N1 imaging, QSM, and DTI remain experimental. While they offer high sensitivity and specificity in controlled research settings, their routine clinical application is currently limited by the need of specialized equipments, lack of standardized acquisition protocols, and the high cost.

#### Magnetic resonance spectroscopy (MRS) of the GABAergic and glutamatergic systems

MRS enables in vivo quantification of neurochemical alterations in PD, including inhibitory (GABA) and excitatory (glutamate) neurotransmitters. Altered GABA/Glu homeostasis within the cortical–basal ganglia–thalamocortical circuit contributes to abnormal firing patterns underlying bradykinesia and rigidity.

Several MRS studies showed reduced GABA concentrations in the thalamus and basal ganglia in PD, reflecting impaired inhibitory control within motor circuits [108]. Conversely, glutamate alterations are more heterogeneous. Some studies demonstrated elevated glutamate levels in the putamen and motor cortex, consistent with excitotoxic stress, whereas others reported reduced glutamate levels depending on disease stage and region [109].

Elevated glutamate in specific regions is associated with worse motor symptoms and may predict the development of dyskinesia, aligning with the role of glutamatergic hyperactivity in levodopa-induced dyskinesia. Overall, the MRS-based neurotransmitter assessment provides a non-invasive approach to mapping circuit-level imbalance beyond dopaminergic mechanisms. Similarly, MRS is strictly an experimental approach. Despite its ability to provide profound mechanistic insights into metabolic and neurotransmitter dysfunctions, its diagnostic accuracy remains insufficiently validated.

#### Energy metabolism crisis

Mitochondrial dysfunction is increasingly recognized as a central pathogenic mechanism in PD, and MRS provides a non-invasive approach to quantify in vivo metabolites related to cellular energy metabolism and oxidative stress [110]. ^31^P-MRS and ^1^H-MRS have been applied to detect high-energy phosphates such as phosphocreatine (PCr) and ATP, as well as anaerobic glycolysis products (e.g. lactate) and antioxidants (e.g. glutathione, GSH), which together reflect the functional integrity of oxidative phosphorylation and redox homeostasis in the human brain[111].

In a study including early and advanced PD patients, combined phosphorus and proton MRS at 3 T revealed bilateral reductions of ATP and PCr in the putamen and midbrain compared with age-matched controls, while low-energy metabolites such as inorganic phosphate remained within normal range. These findings provide strong in vivo evidence that mitochondrial impairment in mesostriatal neurons occurs early and persists in PD [110]. More recently, a multimodal investigation combining putaminal and midbrain ^31^P-MRS with mitochondrial phenotyping in peripheral skin fibroblasts demonstrated that PD patients have impaired oxidative phosphorylation in striatal nerve terminals, altered mitochondrial membrane potential, and dysregulated mitochondrial/lysosomal homeostasis, supporting the hypothesis of systemic mitochondrial dysfunction in early PD [112].

Regarding oxidative stress, a 7 T MRS pilot study detected significantly lower GSH levels in the substantia nigra of PD patients compared with healthy controls, indicating compromised antioxidant defense capacity [113]. In the same cohort, increased magnetic susceptibility (by QSM) suggested elevated iron deposition, consistent with a state of oxidative stress and iron-induced free radical generation. Earlier MRS studies also reported elevated lactate in PD brains, consistent with a shift toward anaerobic glycolysis when mitochondrial oxidative phosphorylation is impaired [111].

Taken together, these metabolic signatures provide evidence of a widespread bioenergetic crisis and oxidative imbalance in PD. While the use of MRS-derived measures to estimate disease risk or monitor therapeutic response remains preliminary, recent multimodal studies suggest that this approach holds promise for mechanistic stratification and evaluation of neuroprotective interventions targeting mitochondrial pathways. A summary of key MRI-based biomarkers of PD is provided in Table 3.

**Table 3:** Structural, metabolic, and clearance profiles of MRI biomarkers

Technique	Pathological target	Clinical utility & key findings
Nigrosome-1 Imaging (SWI/7 T)	Nigrosome-1 loss, iron accumulation, and loss of the “swallow-tail sign”	Achieved high diagnostic accuracy (sensitivity 0.94–1.0; specificity 0.90–0.96) in differentiating PD from healthy controls
QSM	Iron accumulation in the SNc	Correlates with disease duration and MDS-UPDRS III motor scores, serving as a reliable progression marker
Neuromelanin-sensitive MRI	Depletion of neuromelanin-rich dopaminergic and noradrenergic neurons	Differentiated PD with ~ 82% sensitivity/specificity; signal loss progressively declines with disease duration
DTI	Loss of microstructural white matter integrity and FA	Detectable in early stages of PD, though its ability to reflect disease progression remains inconclusive across meta-analyses
MRS	Alterations in GABA/glutamate homeostasis and high-energy phosphates (ATP/PCr)	Reveals early mitochondrial dysfunction (reduced ATP/PCr) and circuit-level neurotransmitter imbalances
DTI-ALPS	Impaired glymphatic and interstitial fluid clearance pathways	Lower ALPS indices are linked to inefficient removal of misfolded αSyn and poor cognitive outcomes

*7 T* 7 Tesla, *ATP* Adenosine triphosphate, *DTI* Diffusion tensor imaging, *DTI-ALPS* Diffusion tensor image analysis along the perivascular space, *FA* fractional anisotropy, *GABA* Gamma-aminobutyric acid, *Glu* Glutamate, *MDS-UPDRS III* Movement Disorder Society-Unified Parkinson’s Disease Rating Scale Part III, *MRS* MR spectroscopy, *PCr* phosphocreatine, *PD* Parkinson’s disease, *QSM* quantitative susceptibility mapping, SNc substantia nigra pars compacta, *SWI* susceptibility-weighted imaging

### PET

Major PET and molecular imaging targets in PD and related synucleinopathies, spanning pathology-anchoring tracers (e.g., αSyn PET), neuroinflammation ligands, and multi-system neurotransmitter mapping, are summarized in Table 4.

**Table 4:** PET and molecular imaging

Category	Targeted biomarkers	Key clinical applications & translational evidence
Direct α-synuclein PET	[^18^F]ACI-12589 and [^18^F]C05–05	[^18^F]ACI-12589 is highly specific for MSA (GCI); [^18^F]C05–05 correlates with PD motor severity and trial target engagement
Neuroinflammation PET	TSPO and next-generation inflammatory ligands	Quantifies glial activation surrounding αSyn inclusions, reflecting pathological activity that may precede progression
Dopaminergic system	Presynaptic (DAT, VMAT2, AADC) and Postsynaptic (D2)	Presynaptic markers distinguish early PD; D2 differentiates PD from MSA/PSP
Other monoaminergic systems	SERT (Serotonin) and NET (Noradrenaline)	Reductions correlate with depression, anxiety, fatigue (SERT) and postural instability or orthostatic hypotension (NET)
Cholinergic system	VAChT (^18^F-FEOBV)	Severe cortical denervation correlates with cognitive impairment, dementia, freezing of gait, and fall risk

*ɑSyn* ɑ-synuclein, *AADC* Aromatic L-amino acid decarboxylase, *DAT* Dopamine transporter, *FEOBV* [^18^F]-Fluoroethoxybenzovesamicol, *GCI* Glial cytoplasmic inclusion, *MDS-UPDRS III* Movement Disorder Society-Unified Parkinson’s Disease Rating Scale Part III, *MSA* Multiple system atrophy, *NET* Noradrenaline transporter, *PET* Positron emission tomography, *PSP* Progressive supranuclear palsy, *SERT* Serotonin transporter, *TSPO* Translocator protein, *VAChT* Vesicular acetylcholine transporter, *VMAT2* Vesicular monoamine transporter 2

#### αSyn pathology: build-up and spread

The abnormal misfolding, aggregation, and propagation of αSyn represent the central pathological cascade driving PD and related synucleinopathies. Molecular imaging approaches, particularly αSyn-targeting PET, have rapidly advanced in recent years, transforming αSyn from an inaccessible intracellular protein into a quantifiable in vivo biomarker. Yet, αSyn imaging remains uniquely challenging due to the extremely low abundance of aggregates in PD, the predominantly intracellular localization, and the strain-dependent conformational differences across PD, DLB, and MSA [114, 115].

Second- and third-generation αSyn PET tracers are emerging to overcome these barriers. [^18^F]ACI-12589 provides the most robust evidence to date for in vivo detection of αSyn pathology, demonstrating high-affinity and high-selectivity binding to oligodendroglial cytoplasmic inclusions in MSA and achieving scan-to-autopsy validation of tracer–pathology correspondence [116]. However, a critical limitation is its weak binding to Lewy bodies in typical PD patients. This finding underscores a profound difference in the structural biology between PD and MSA: Lewy bodies have a fibril density approximately 100 times lower and a looser structure compared to glial cytoplasmic inclusions (GCI). The minimal uptake of [^18^F]ACI-12589 in typical PD highlights the necessity for subtype-specific tracer design [117].

In contrast, the benzothiazole-derived ligand [^18^F]C05-05 represents a key milestone for PD imaging: it successfully visualizes midbrain αSyn deposition, with substantia nigra uptake correlating with the MDS-UPDRS III motor severity [118]. This establishes αSyn PET as a promising candidate biomarker not only for disease staging and phenotypic stratification but also for quantifying target engagement in αSyn-lowering therapeutic trials [119]. However, a significant challenge is the off-target binding, particularly cross-reactivity with AD pathologies (Aβ and tau). This complicates image interpretation in older PD patients, who often have co-existing AD pathology, necessitating “subtraction” analysis in conjunction with Aβ-PET.

Beyond direct visualization of aggregates, complementary molecular imaging approaches capture the brain’s secondary responses to αSyn accumulation. Microglial and astrocytic activation, long recognized as amplifiers of αSyn pathology, can be quantified using inflammation-targeted PET tracers. TSPO and next-generation inflammatory ligands reveal elevated binding in PD within the substantia nigra, striatum, and associative cortices, patterns consistent with the histopathological evidence of glial reactivity surrounding αSyn inclusions [120, 121]. As such, inflammation PET serves as a microenvironmental biomarker, reflecting pathological activity that may precede or accelerate clinical progression.

A complementary view on the build-up and spread of αSyn pathology comes from MRI-based markers of impaired glymphatic and interstitial fluid clearance. DTI-ALPS (diffusion-tensor analysis along perivascular spaces) and assessment of enlarged perivascular spaces (PVS) suggest compromised waste-clearance pathways in PD [122, 123]. Reduced ALPS indices and increased PVS burden imply inefficient removal of misfolded proteins and metabolites, which may facilitate αSyn accumulation and promote its dissemination along structural and functional networks [124]. These MRI signatures therefore operate as systems-level biomarkers, capturing clearance failure that interacts with molecular pathology.

Together, αSyn PET, inflammation PET, and clearance-related MRI form an emerging multimodal biomarker framework that maps the initiation, amplification, and propagation of αSyn pathology. With continuing improvement of tracer specificity and increasing longitudinal studies, these modalities will enable earlier and more accurate diagnosis, refined biological subtyping across synucleinopathies, and rigorous evaluation of therapeutic efficacy in clinical trials. Ultimately, molecular imaging of αSyn build-up and spread represents one of the most transformative frontiers in biomarker research for PD.

#### Dopamine system

Dopaminergic neuronal loss in the substantia nigra is a hallmark pathology of PD, resulting in striatal dopamine deficiency. PET and Single-Photon Emission Computed Tomography (SPECT) allow in vivo assessment of presynaptic dopamine terminal integrity by quantifying DAT, aromatic L-amino acid decarboxylase (AADC) activity, vesicular monoamine transporter 2 (VMAT2) expression, and dopamine receptor binding. DAT imaging with both PET (e.g., ^11^C-RTI-32, ^18^F-FP-CIT) and SPECT tracers (e.g., ^123^I-FP-CIT, ^99^mTc-TRODAT-1) consistently shows reduced uptake in the caudate and putamen of PD patients compared with controls [125, 126]. In contrast to experimental PET tracers, DAT imaging (e.g., ^123^I-FP-CIT SPECT or DaTscan) is a well-established, regulatory-approved clinical tool. Decreases in ^18^F-FP-CIT/^123^I-FP-CIT uptake and TRODAT-1 ratios provide high diagnostic accuracy for early PD [127, 128], and DAT SPECT effectively distinguishes PD from essential tremor (ET) (sensitivity 91%, specificity 100%) [129]. Nonetheless, discrimination between PD and atypical parkinsonian syndromes (APS) remains limited, although deep-learning approaches demonstrate improved performance [130]. Longitudinal DAT imaging reveals an annual striatal decline of 11% and follows an exponential loss pattern in early–moderate PD [131, 132].

^18^F-Dopa PET assesses AADC function and correlates with nigral cell loss [133, 134]. PD patients exhibit early, marked reductions in dorsal putamen uptake, with relative sparing of the ventral putamen until later disease stages [135]. ^18^F-Dopa kinetic parameters such as* K*_occ_ and Effective Distribution Volume Ratio (EDVR) show excellent diagnostic accuracy for PD from controls (> 97% sensitivity) [136], while specific regional ratios improve differentiation from APS [137].

VMAT2 imaging using ^11^C-DTBZ or ^18^F-FP-DTBZ reflects monoaminergic terminal integrity and correlates with disease severity [138]. Posterior putamen binding ratios reach 100% sensitivity and specificity for separating PD from healthy subjects [139]. Younger-onset PD shows greater initial DTBZ loss but slower subsequent decline, and reduced DTBZ uptake is also detectable in isolated RBD [140].

Postsynaptic dopamine receptor imaging shows relatively preserved D1 binding but early alterations in D2 receptors [141]. PD is associated with an initial upregulation of D2 receptors, which reverses to downregulation after approximately 4.4 years. In contrast, PSP and MSA demonstrate markedly lower striatal D2 receptor binding, aiding in differential diagnosis [142].

#### Serotonergic and noradrenergic system

Non-dopaminergic monoaminergic degeneration represents a key pathological component of PD and contributes significantly to symptoms such as depression, anxiety, fatigue, gait dysfunction, and sleep disorders. Serotonin (5-HT) neurons arising from the dorsal raphe and noradrenergic (NA) neurons originating in the locus coeruleus, exhibit early and progressive degeneration in PD. PET ligands targeting serotonin transporters (SERT) and noradrenaline transporters (NET) provide in vivo evidence for this neuronal loss.

SERT imaging using radiotracers such as ^11^C-DASB has consistently demonstrated widespread SERT binding reductions in cortical, limbic, and brainstem in PD patients, even at early stages of disease [143]. Reduced SERT binding correlates with non-motor symptoms including depression, anxiety, and fatigue [144]. Longitudinal studies suggest that the SERT decline may precede the onset of motor symptoms, aligning with Braak staging and supporting a multisystem neurodegenerative model of PD.

Similarly, noradrenergic dysfunction can be assessed using NET ligands such as ^11^C-MeNER, which have shown significantly reduced NET binding in the locus coeruleus, thalamus, and hypothalamus [145]. The degree of NET loss is associated with postural instability and gait difficulty, orthostatic hypotension, and RBD [146]. Because SERT and NET deficits occur earlier than dopaminergic changes, these markers hold promise for prodromal PD stratification.

Collectively, these PET-based measures provide complementary readouts of neurotransmitter integrity, metabolic dysfunction, protein aggregation, and neuroinflammatory responses.

#### Vesicular acetylcholine transporter (VAChT) PET in the cholinergic system

Cholinergic degeneration is another hallmark of PD and is increasingly recognized as a major contributor to cognitive impairment, gait disturbance, olfactory dysfunction, and falls. PET imaging of VAChT, most commonly using ^18^F-FEOBV, allows measurement of presynaptic cholinergic terminal integrity.

Studies using ^18^F-FEOBV PET have revealed severe and widespread cortical cholinergic denervation, particularly in the temporal and parietal regions, in PD patients with mild cognitive impairment and dementia [147]. Cholinergic loss in the basal forebrain and pedunculopontine nucleus also correlates with postural instability, freezing of gait, and fall risk [148]. Notably, PD and DLB differ in the severity and distribution of cholinergic loss, suggesting utility for differential diagnosis [149].

Moreover, cholinergic deficits interact with dopaminergic dysfunction: reduced VAChT binding in the striatum predicts poor motor response to dopaminergic medications, supporting a multisystem framework for PD motor symptoms.

#### Imaging of Aβ and tau co-pathology

While PD is primarily defined by synucleinopathy, protein co-pathology is highly prevalent and exerts a profound impact on the clinical phenotype, particularly cognitive decline [150]. Aβ PET imaging using tracers such as ^18^F-Florbetapir, ^18^F-Flutemetamol and ^11^C-PiB, is an important tool for identifying Alzheimer’s-type co-pathology [151]. The presence of cortical Aβ deposition in PD and PD with mild cognitive impairment is a strong predictor of faster cognitive decline and a higher risk of conversion to PD dementia or DLB [152]. Assessing this co-pathology via PET not only aids in the differential diagnosis of distinct cognitive profiles, but is also increasingly used as a prognostic biomarker and an enrichment tool for clinical trials targeting cognitive impairment in synucleinopathies.

## Overview of digital biomarkers

Digital biomarkers and traditional biomarkers (e.g., imaging and biofluid) pursue similar clinical goals, including disease risk identification, disease characterization, therapeutic efficacy evaluation, and progression tracking [153–156]. However, they differ fundamentally in sources, sensing, construction, and evaluation systems. Digital biomarkers are becoming a distinct methodological system in PD.

### A conceptual framework for digital biomarkers in PD

#### Source

Traditional biomarkers are typically obtained from standardized, static and short-duration assessments (e.g., posture, a single movement) in clinical settings and target biological entities (e.g., brain structure, brain function, cellular components, and protein molecules). In contrast, digital biomarkers arise from task-based or naturalistic behaviors across in-clinic (standardized, low-noise, controlled against gold standards), home (high-frequency, continuous, ecological validity), and outdoors (natural load, real-world environment) scenarios. The targets of observation cover clinical phenotypes such as movement, behavior, facial expression, and speech as well as general physiology (e.g., heart rate) associated with these phenotypes [154, 157, 158,159].

#### Sensing

Traditional biomarkers rely on imaging or biofluid testing to probe internal pathology; whereas digital biomarkers leverage multimodal sensing technologies such as wearable Inertial Measurement Unit (IMUs), machine vision, acoustic analysis, wireless sensing, device interaction, eye-tracking, and photoplethysmography (PPG), to capture high-dimensional dynamic signals across body sites (e.g., upper limbs). Digital biomarkers enable phenotyping of gross motor activity, fine movements, natural behaviors, complex speech, and general physiological domains [154, 160–166].

#### Construction

Traditional biomarkers typically rely on single-dimensional or independent features after standardization, normalization, and integration with population reference values. Recently, they have gradually shifted toward combination into composite indices by machine learning (ML) or deep learning (DL). In contrast, digital biomarkers typically rely on the construction of specific models and formation of composite indices by combining artificial intelligence (AI) algorithms with multimodal high-dimensional features for specific clinical outcomes and tasks. Therefore, digital biomarkers are more appropriately viewed as model-derived composite indices rather than single stand-alone signals.

#### Interpretability

Traditional biomarkers have more direct correlations with disease mechanisms. In contrast, digital biomarkers primarily quantify observable clinical phenotypes and then correlate them with underlying mechanisms by referencing known pathophysiological pathways and clinical experience, thereby supporting interpretability across levels of models, features, mechanisms, and clinical aspects.

#### Evaluation

Traditional biomarkers emphasize sensitivity, specificity, reliability and validity, whereas digital biomarkers additionally require responsiveness, reproducibility, cross-device consistency, ecological generalizability, fairness, and long-term stability [158, 167–169.

#### Regulatory acceptability

This evaluation philosophy is also reflected in regulatory systems worldwide. As digital biomarkers are still evolving but show clear advantages for multidimensional characterization of disease dynamics, regulatory agencies in different countries have established relevant provisions, from safety and effectiveness to the rigor and compliance of their use as digital endpoints, with continuing optimization and exploration. However, to date, fully validated examples in global, multicenter, multi-ethnic settings remain limited and largely preliminary [170–174]. The regulatory acceptability primarily refers to the methodological maturity in evidence generation, validation routes, and endpoint definition, rather than formal regulatory approval by a single agency.

**Fig. 4:**
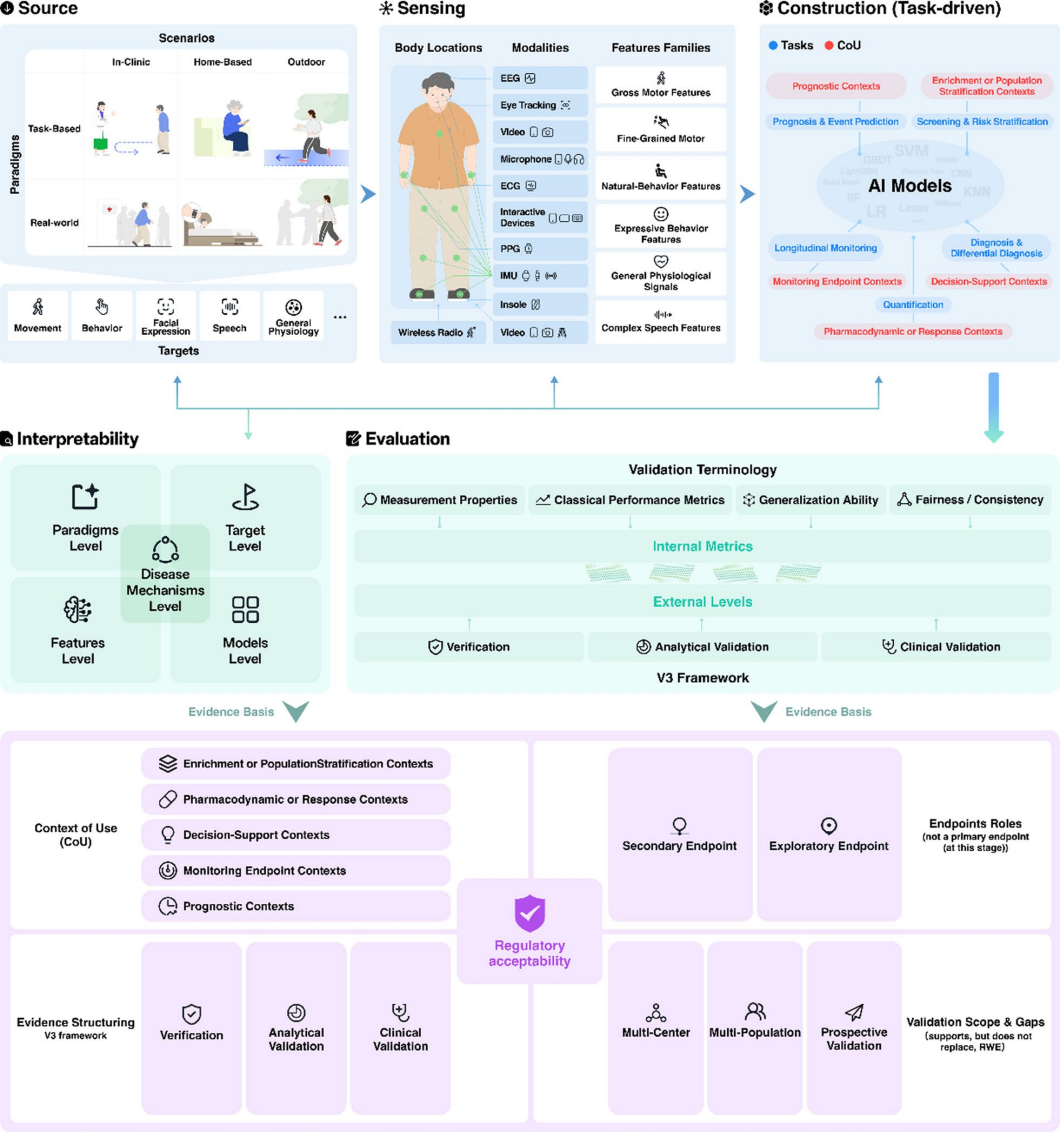
A conceptual framework for digital biomarkers in Parkinson’s disease, consisting of source, sensing, construction, interpretability, evaluation, and regulatory acceptability. *SVM* Support vector machine, *GBDT* Gradient boosting decision tree, *LightGBM* Light gradient boosting machine, *HSMM* Hidden semi-Markov model, *CNN* Convolutional neural network, *KNN* k-nearest neighbors, *RF* Random forest, *LR* Logistic regression, *Lasso* Least absolute shrinkage and selection operator, *XGBoost* eXtreme gradient boosting

Here, we propose a complete methodological framework for digital biomarker development. The framework includes source (paradigm–scenario–target), sensing (technology–body site–features), construction (AI models oriented to specific tasks and outcomes), interpretability, evaluation systems, and regulatory acceptability (Fig. 4). To avoid conceptual ambiguity, we distinguish between two related but distinct regulatory concepts. The first is the regulatory authorization or clearance of a digital health technology including software as a medical device for a specified intended use. This type of review primarily concerns whether the technology is safe, performs as intended, and is appropriate for its claimed clinical use. The second is the regulatory acceptability of a digital biomarker for a defined context of use in drug development, such as patient enrichment, disease monitoring, or use as a clinical trial endpoint. Evidence frameworks such as V3 (verification, analytical validation, and clinical validation), together with task-specific evidence mapping, can help structure the evidence package for regulatory review. However, these frameworks do not by themselves imply that a digital biomarker is acceptable for diagnostic use or as a primary efficacy endpoint. In PD, most digital biomarkers are therefore currently better positioned for enrichment, monitoring, and exploratory or secondary endpoint applications, while broader use as diagnostic tools or primary endpoints will require further prospective, multi-center validation.

### Sources and phenotypic space

#### Task-based vs real-world paradigms

Digital biomarkers in PD can first be understood from the perspective of “signal sources”. At the measurement-paradigm level, they mainly originate from two modes: task-based paradigms and unconstrained real-world paradigms.

In task-based paradigms, patients complete standardized motor tasks and other specific paradigm assessments (e.g., speech or cognitive tasks) under controlled or semi-controlled conditions, during which multiple digital sensing modalities (e.g., wearable devices, microphones, cameras, and other systems) synchronously collect signals related to external manifestations such as movement, behavior, and speech. Examples include device-based Timed Up and Go, straight-line walking and turning, standardized standing and balance tests, MDS-UPDRS III scale paradigms (e.g., finger tapping), spiral or line drawing, standard eye saccades, and speech tasks such as sustained phonation or reading aloud [161, 167, 175–178].

In contrast, real-world paradigms collect data during daily activities (e.g., walking and transfers, housework, sleep, spontaneous speech and communication, and daily interactions with devices such as smartphones and keyboards) [157, 168, 179, 180]. A waist-worn accelerometer can function as an objective “motor diary”, deriving ON/OFF and dyskinesia biomarkers from continuous recordings [181]. Nocturnal actigraphy and combined sensor monitoring can quantify sleep behaviors ecologically, including isolated REM sleep behavior disorder [157, 165].

Overall, task-based paradigms rely on controlled, repeatable trials to extract high signal-to-noise indices targeting specific motor or cognitive pathways, whereas real-world paradigms prioritize ecological validity and phenotypic diversity, better reflecting the real-world functional status. Together, they capture maximal capability under standardized conditions and daily functional performance in real life, respectively, and are complementary.

#### Clinical–home–outdoor continuum

From the perspective of measurement environments, PD digital biomarkers are collected across scenarios ranging from standardized laboratories to naturalistic community environments. In-clinic or laboratory assessments emphasize standardization, controllable noise, and alignment with clinical gold standards: patients perform predefined tests under supervision using calibrated sensors, video, microphones, or other devices such as tablets, supporting technology validation and algorithm development [166, 182–184]. Home-based deployment prioritizes high-frequency, longitudinal, and ecologically valid sensing [157, 167]. In remote settings, smartphones can administer brief tasks (walking, tapping, speech); their multisensors capture multimodal motor and speech data for transmission and analysis via the Internet of Medical Things (IoMT, a network of medical/health devices and platforms enabling secure data transfer and integration), enabling automated identification and screening of subtle early-PD symptoms [185]. Compared with outpatient follow-up, these platforms have increased assessment frequency and ecological validity but demand robust infrastructure, particularly privacy protection and patient digital literacy. Community and outdoor assessments place biomarkers in natural behavior of daily living [158, 186]. From regulatory and implementation perspectives, clinic/lab settings enable early validation of technical feasibility and measurement properties [187], whereas free-behavior assessments at home/community/outdoors are primary data sources for real-world research and extended endpoints in clinical trials [158]. Together, they form a continuum for digital biomarkers from “feasibility” to “applicability” to “regulatory acceptance” [173, 188].

#### Phenotypic dimensions

Gross motor features capture global impairment (bradykinesia, gait, postural stability) [153, 175, 189], whereas fine-grained motor signals characterize tremor, fine hand movements, and subtle limb movements [167, 182, 190]. Natural behavior signals can be detected using long-term wearable devices and environmental sensors to quantify activity, circadian rhythms, daily-walking, home-activity, and sleep patterns, reflecting functional status and compensatory strategies in real life [157, 186]. Active and passive facial measures quantify changes in expressive behaviors such as facial expressions, blinking, and gaze shifts, indicating subtle phenotypic features such as “masked face”, reduced emotional expression, and visuo–gait coordination [154, 191, 192]. Complex speech signals from structured tasks or natural conversations provide acoustic–linguistic information covering phonation, articulation, prosody, and language use, enabling objective quantification of hypokinetic dysarthria (e.g., degraded voice quality) and supporting early detection and phenotypic differentiation [154, 180, 184]. General physiological signals mainly originate from wearable electrocardiography (ECG)/heart rate sensors and polysomnography (PSG) devices [159, 193]. Overall, these multidimensional targets jointly constitute the basis of PD digital biomarkers. Importantly, these signals and phenotypic dimensions should be regarded as digital phenotypes rather than digital biomarkers before fit-for-purpose validation.

### Sensing technologies and feature categories

#### Sensing modalities

IMUs worn on the limbs, trunk, and head remain the primary tools for gait and posture assessment, particularly for targets requiring continuous monitoring. They support quantitative assessment and continuous monitoring of tremor, bradykinesia, gait impairment, postural and balance impairments, dyskinesia, and on–off phenomena, and have been widely used in structured tests and daily life [162, 182, 190, 194, 195]. Single-wrist accelerometers have been shown to approximate more complex multi-sensor configurations for tracking motor fluctuations, providing a low-burden solution suitable for home environments [190, 196].

Camera-based computer vision methods complement IMU approaches: videos collected in hospitals, homes, or semi-natural corridor settings have been used to reconstruct three-dimensional hands and body pose, enabling finger-tapping tests, bradykinesia scoring, and contextualized gait assessment without wearable sensors [160, 183, 197].

Speech sensing, using microphones in smartphones, headphones, or dedicated devices, typically uses quantitative acoustic features and modeling to support common tasks, including (1) speech sensorimotor control mechanisms, phenotypic differentiation, and treatment response, (2) cognition–speech coupling and cognitive-state classification, (3) automated identification and longitudinal progression monitoring [154, 184, 198–202]. For example, key speech dimensions under tasks with different cognitive loads can distinguish PD with different cognitive states from healthy controls [200]. Recently, self-supervised speech foundation models have been explored for feature extraction or adapted for speech phenotyping of PD. These models may improve robustness in noisy and small-data settings, but their learned high-dimensional speech representations are often less interpretable than conventional acoustic features. Therefore, model explanation strategies are needed when these representations are proposed as digital biomarkers or clinical trial endpoints.

Wireless sensing enables contactless monitoring. For example, a low-power radio sensor in a participant’s bedroom can extract nocturnal breathing signals by analyzing radio-frequency reflection signals during sleep, supporting AI-based PD detection, disease severity assessment, and disease-course progression tracking [165]. This approach enables continuous contactless monitoring at home and can better balance between usability and privacy.

Smartphones and tablets serve as multimodal sensing and interaction terminals, integrating IMU, cameras, touch input, and audio to support unified digital assessment platforms [163, 166, 185]. Eye-tracking technology quantifies characteristic eye-movement patterns across neurodegenerative disorders (e.g., AD/mild cognitive impairment), reveals significant between-group differences, and may provide potential digital biomarkers for disease differentiation [203]. In PD, it also characterizes oculomotor function under different stimuli and task conditions [161]. In the PPMI cohort, some participants wore multisensor smartwatches integrating PPG to passively collect digital measures such as activity and sleep; these features correlate with non-motor symptom scales for cognition, autonomic nervous system, and activities of daily living, suggesting that the multimodal features derived from technologies such as PPG may serve as digital biomarkers for remote monitoring of PD non-motor symptoms [164].

Together, these modalities, including IMU, vision, speech, wireless sensing, and physiological monitoring, illustrate the breadth of sensing strategies underpinning PD digital biomarkers.

#### Body location strategies

Body-location sensing technologies can span the body. Head- and trunk-mounted sensors capture balance, postural sway, and overall axial control [162, 204]. Eye-movement and eye–gait fusion systems, combining visual-cue tasks with wearable eye tracking systems, IMU, and AI computer vision system, assess visuo–gait coordination patterns in PD (with and without freezing of gait [FoG]). They add value in daily/real-world settings for gait feature extraction, fall-risk assessment, and subtype stratification (e.g., FoG vs non-FoG) [205, 206]. Video and audio monitoring of the facial and perioral regions can quantify facial masking and dysarthria [154, 191, 198, 199]. Upper-limb (including distal hand) function is assessed using IMU and/or video-based pose estimation during tasks such as finger tapping and hand rotation, or via spiral drawing on smart touchscreens [160, 162, 178, 183]. Wrist-worn sensor devices are widely used in structured tests and continuous home monitoring to record tremor, bradykinesia, gait, motor complications, and overall activity levels [182, 189, 190]. Waist- and lower-limb sensors (e.g., ankle or in-shoe IMUs) can record activity levels, gait initiation, gait stance and swing phases, turning, and FoG events in laboratory and real-world conditions [163, 195]. Based on patient burden and adherence, body-location selection should match assessment purposes [207, 208].

#### Feature categories

Based on the body locations, representative features can be extracted (Table 5). Common gross motor features include spatiotemporal ambulatory gait parameters, postural sway indices, arm-swing kinematic, turning dynamic, and sit-to-stand/stand-to-sit transitions measures [153, 175, 195]. Fine motor features primarily reflect tremor and micro-motor abnormalities, including tremor amplitude and its spectral distribution, finger-tapping speed and rhythmicity, and spiral-drawing smoothness and micrographia [167, 178, 182, 190].

**Table 5:** Overview of feature families, targets, sensing technologies, sensing locations, and feature examples

Feature categories	Target	Sensing technology	Sensing location	Feature examples	References
Gross motor feature	Movement	Wearable IMU	Ankles, Wrists, Lumbar, Sternum	Gait speed, Cadence, Coronal range of motion, Sway area radius [coronal], etc	[153]
			Thighs, Shanks, Chest, Hands, Waist, Feet	Stride length, Arm-backward swing Max, Stride velocity asymmetry, Stand to Sit-trunk-min lean angle, Lumar-sway max, 180° Turn-steps (#), etc	[175]
			Lumbar, Feet	Average turning angle, Average turn peak velocity, Average turning duration, Gait speed, etc	[195]
			Thighs, Shanks, Chest, Hands, Waist, Feet	MAS step length, MAS cadence, MAS shank forward swing maximum, Turning-average duration, SiSt-trunk sagittal peak velocity, StSi-trunk sagittal peak velocity, etc	[162]
			Shoes, Lower back	Gait speed, Cadence, Stride length, Stride time variability, etc	[155]
			Wrists, Feet, Shanks, Sternum, and Lumbar spine	Gait speed, Stride length, Foot strike angle, Stride time, and Stride time variability, etc	[206]
		Wearable IMU (Smartwatch)	Wrist	Arm swing acceleration, Arm twist amplitude, etc	[189]
	Behavior	Wearable video glasses, Wearable IMU	Waist, Eye	Step time, Swing time asymmetry, Stairs/steps within the immediate path, etc	[205]
Fine motor features	Movement	Wearable IMU	Feet	Power in the “freeze band” (3.5–8 Hz), Power in the “locomotor band” (0.5–3 Hz), etc	[195]
			Upper arm and Forearm	MeanT, MeanV, etc	[194]
		Eye tracking	Eye	Saccade latency, Velocity, Duration, and Amplitude, etc	[161]
				Saccade count, Duration, Distance, Velocity, and Latency of prosaccade or antisaccade, etc	[203]
		Tablet (Touchscreen interaction)	Hand	Smoothness of the spiral, etc	[178]
			Hand, Finger	Movement speed, Movement amplitude, etc	[166]
		Smartphone (Touchscreen interaction)	Finger	Kurtosis, Number of peaks, Partial autocorrelation, etc	[167]
		Video	Body, Hand	Joint collection distances, Two scale motion, etc	[160]
		Sensor insoles	Plantar surface	Pressure distribution, etc	[163]
		Video (Camera)	Upper Limb, Lower limb	Absolute jerk, Absolute acceleration, etc	[183]
		Mobile infrared eye-tracker	Eye	Accade frequency, Duration, Peak velocity, amplitude, and Fixation duration, etc	[206]
		Smartphone (Gyroscope, Accelerometer, and Touchscreen interaction, Microphone)	Body, Fingers, Mouth	Mel-Frequency cepstral coefficient, Zero-crossing rate of the time signal, Root mean square signal frame energy, etc	[185]
	Movement, Behavior	Wearable IMU (Smartwatch)	Wrist	Tremor displacement, Existence of a sharp peak in our FFT within the 3–7 Hz bins, Motion strength, Spectral entropy, etc	[190]
		Wearable IMU	Wrist	Signal entropy, Dominant frequency, Spectral flatness, etc	[182]
			Wrists and Ankles, Lower back	Total power in the 0.5- to 10-Hz band, width of the dominant frequency, etc	[158]
	Speech	Microphone	Mouth	First formant frequency acuity, Vocal fundamental frequency, etc	[198]
				Harmonics-to-noise ratio, Cepstral peak prominence, Resonant frequency attenuation, etc	[199]
				Jitter, The number of voiced segments per second, The average and standard deviation of the duration of voiced, unvoiced, and pauses, etc	[200]
				Cepstral peak prominence, MFCC, Recurrence period density entropy and correlation dimension, etc	[202]
Natural-behavior features	Behavior	Wrist-worn triaxial accelerometer	Wrist	Number of nocturnal awakenings, Length of uninterrupted sleep, Overall sleep hours, etc	[157]
		Wristband	Wrist	Sleep duration, Number of wakeups, Ratio of time asleep to total sleep, Total length of restless, etc	[163]
		Smartwatch (with PPG)	Wrist	Step count, Walking minutes, Rapid eye movement time, etc	[164]
Expressive behavior features	Facial expression	Video (Camera)	Face	Eye-blinking time, Mouth height/width variance, Mouth angle variance, Mouth-to-eye distance variances, Peri-oral area movement variances, etc	[154]
				Sizes and muscle movement between facial landmarks, etc	[191]
				Facial expression amplitude, Shaking of small facial muscle groups, etc	[192]
Complex speech features	Speech	Microphone (Smartphone)	Mouth	Reading time, Phonetic score, Pause percentage, Voice volume variance, Average pitch, and Pitch variance, etc	[154]
		Microphone	Mouth	Rate of verbs, Case particles (dispersion), Verb utterances, Rate of common noun utterances, Proper noun utterances, and Filler utterances, etc	[180]
		Head-mounted condenser microphone	Mouth	Maximum phonation time, Relative loudness of respiration, Harmonics-to-noise ratio, and Cepstral peak prominence, etc	[184]
General physiological signals	General physiology	ECG	Chest	Heart rate, Heart rate variability, Event-related heart rate change, etc	[193]
				Heart rate variability: NN heartbeat interval, Standard deviation of NN, High-frequency power normalized units, low frequency to high-frequency ratio, etc	[159]
		wristband	Wrist	Heart rate, Galvanic skin response, and Skin temperature data, etc	[163]
		Smartwatch (with PPG)	Wrist	Pulse rate, Mean RMSSD (heart beat), Median RMSSD, RMSSD variance, etc	[164]

*IMU* Inertial measurement unit, MAS More affected side, *LAS* Less affected side, Sist Sit to stand, *Stsi* Stand to sit, *PPG* Photoplethysmography, *ECG* Electrocardiography, *FFT* Fast Fourier Transform, *MFCC* Mel-frequency cepstral coefficients, *NN* Normal-to-normal intervals, *RMSSD* Root mean squared successive differences

Natural-behavior features capture diurnal activity rhythms, sedentary bouts, nocturnal activity, and context-dependent gait adaptations, reflecting real-world functional status and compensatory strategies [157, 165]. Expressive behavior features (facial expressiveness richness, blink rate, and facial-keypoint-based geometric and muscular movement) quantify manifestations such as facial masking and affective changes [154, 191, 192]. Complex speech features span voice quality, articulatory precision, speech rate rhythm, and prosodic structure; they comprehensively reflect changes in speech control, supporting identification of PD phenotype differentiation and quantification of impairment severity [154, 180, 184].

General physiological signals from ECG, PPG, and PSG provide measures of heart rate variability, autonomic nervous system function, and sleep physiology, supplementing motor phenotypes [159, 193]. Cardiovascular features (e.g., heart rate) reflect autonomic responses related to motor intention in laboratory-evoked gait tasks and distinguish FoG from voluntary stopping, resolving the limitation that motion sensors alone often cannot distinguish FoG events [193]. Nocturnal PSG simultaneously obtain non-motor physiological indices such as sleep architecture and heart rate variability to quantify non-motor symptoms such as sleep disorders. In PD, reduced REM density is associated with impaired gait and motor ability and can be influenced by sustained-release dopaminergic medications in the evening, suggesting that the REM-related sleep alterations reflect neuroregulation abnormalities beyond motor function [159]. These feature categories provide basis for constructing digital biomarkers. However, AI-based mining and multi-approach validations are required before they can be used as clinical trial endpoints or for supporting clinical decision-making [209, 210].

### From signal to digital biomarkers: AI construction

#### Classical ML vs DL

ML and DL are typically used in PD digital biomarker systems. The classical ML pipeline starts from manually designed (“handcrafted”) features from IMU, videos, audios, or physiological signals. These features include time-domain and frequency-domain descriptors, gait spatiotemporal parameters, variability and entropy indices, and simple composite scores. They are input to models such as support vector machines, random forests, and gradient boosting trees to perform tasks such as differentiation of PD versus controls, differential diagnosis, and regression of symptom severity [158, 175, 182]. In contrast, DL directly learns from raw signals (IMU time series, audio waveforms, video frames) using models such as convolutional networks, recurrent architectures (including long short-term memory networks), temporal convolutional networks, GNN (graph neural networks), and Transformers; however, they often face problems of overfitting, requirement of large amounts of data, and insufficient interpretability [185, 197, 211, 212]. The GFN (graph fusion neural network) architecture combined with footstep pressure maps and video recording showed promising performance in FoG detection [197]. In the IoMT-based frameworks, deep learning models can integrate IMU signals, tapping-task data, and voice signals to support early PD detection [185].

#### Task-driven modeling

Digital biomarker modeling is typically task- and endpoint-driven, and multimodal fusion strategies are constantly employed. At the sensing level, IMU, video, speech, respiration, and heart rate modalities can be used simultaneously or separately. At the feature level, handcrafted features or deep representations are aligned for joint modeling. At the decision level, classical ML, DL, or ensembles are combined with outputs from multiple submodels to improve the robustness and generalization in real-world settings [185].

### Clinical tasks and digital endpoints

#### Screening & risk stratification

For early screening and risk prediction, studies using wearable devices, video-based assessments, smartphones, and speech tasks have demonstrated that digital biomarkers enable low-cost, scalable risk stratification and case-finding in self-selected or enriched at-risk populations in clinic [154, 157, 213]. A speech-based study proposed a federated learning framework (FedOcw) for automated PD detection across diverse linguistic and institutional settings. This framework improved cross-lingual knowledge transfer and convergence stability while protecting privacy; and it outperformed traditional methods in diagnostic accuracy [213]. A UK Biobank wrist-worn accelerometer study showed that the accelerometry features can serve as potential digital biomarkers for prodromal and diagnosed PD; ML models based on these data outperformed models based separately on genetics, lifestyle, blood indicators, and traditional prodromal signs in precision-recall performance [157]. Overall, current evidence supports digital biomarkers as tools for risk enrichment, prioritization for formal clinical assessment, and clinical-trial recruitment, but not yet as stand-alone instruments for population-wide screening in asymptomatic individuals.

#### Diagnosis & differential diagnosis

Digital biomarkers—including tremor patterns, gait and balance features, speech features, and REM sleep behaviors, have been used for distinguishing PD, healthy individuals, ET, and other movement disorders in clinical and remote settings [157, 167, 175, 214–216]. In the PD-versus-ET scenario, instrumented Timed Up and Go (TUG) tests using wearable IMUs extract gait and postural-transition features and classify participants with ensemble ML, achieving good discrimination of early PD versus ET (AUC 0.91) [175]. Wearable IMU-derived TUG kinematics can also distinguish tremor-dominant (TD) and postural instability/gait difficulty (PIGD) PD from healthy controls respectively at early stages (AUC 0.7–0.9) [162]. For speech, Yokoi et al. used natural language processing on spontaneous speech and found systematic differences between PD and controls in morphemes counts, part-of-speech distributions, and syntactic features; ML models distinguished PD from healthy individuals with over 80% accuracy [180].

#### Quantification

In quantitative assessment, digital biomarkers can approximate or complement MDS-UPDRS motor scores to evaluate the severity of motor symptoms (e.g., tremor, bradykinesia, gait and postural stability) and non-motor symptoms (e.g., sleep, cognition, and mood) [189, 200, 217–219]. Zhu et al. developed a machine-vision and machine-learning framework to score all MDS-UPDRS III subitems and validated its feasibility in 2610 videos from 149 PD patients; most subitem models showed good-to-excellent agreement with clinical ratings, suggesting that the video-derived objective features can complement subjective scales for motor assessment [219]. A video-based finger-tapping system (e.g., FastEval Parkinsonism) using MediaPipe to extract 3D hand keypoints from finger-tapping videos, quantified MDS-UPDRS finger tapping severity with the best-performing model achieving an acceptable accuracy of 88%, supporting short-video quantification of bradykinesia [220]. A large crowdsourcing study showed that smartphone and wrist-worn sensor features predict the severity of tremor, dyskinesia, and bradykinesia with high accuracy (e.g., bradykinesia area under the precision–recall curve (AUPR) ≈ 0.95), enabling objective, population-scale symptom scoring that can be comparable or more sensitive [218].

#### Longitudinal monitoring

For continuous monitoring, digital biomarkers can dynamically track core motor symptoms (e.g., tremor, bradykinesia, gait, and posture), non-motor symptoms (e.g., sleep), and motor complications (e.g., motor fluctuations and dyskinesia) objectively at high frequencies over days to months [157, 181, 182, 190, 195, 196]. An FoG detection algorithm using foot- and waist-worn inertial sensors achieved moderate-to-high agreement in the number of FoG episodes with expert laboratory ratings. During 7-day home monitoring, the percent time spent freezing and the variability of time spent freezing, detected with this method, distinguished patients with and without FoG [195]. A hierarchical modeling approach using unilateral wrist-worn accelerometer enables real-world monitoring of resting tremor and bradykinesia; the sensor-derived indices showed good-to-high agreement with clinical symptom ratings and detected treatment-related motor-state changes [182]. Powers et al. proposed and validated a smartwatch-based monitoring system to continuously quantify tremor and dyskinesia fluctuations in PD; the outputs were highly consistent with clinical assessments and reflected treatment responses, showing potential for individualized medication management and long-term follow-up [190].

#### Prognosis & event prediction

Prognosis and event prediction models mostly use sensing technologies to capture digital features to predict medication treatment effects, trajectories of motor and non-motor symptoms progression, and risk of specific outcomes such as falls and severe motor complications (e.g., motor fluctuations), supporting individualized treatment decision-making and prognostic stratification [156, 176, 221, 222]. One study used short-duration in-clinic wearable IMU assessments (2-min walk, 30-s postural sway) to predict first-fall risk in PD within 24 and 60 months [222]. Features extracted from wearable and environmental sensors have also been used to quantify efficacies of invasive and noninvasive neuromodulation interventions (e.g., deep brain stimulation and transcranial magnetic stimulation), although current work is largely descriptive rather than strictly predictive [155, 223].

#### Mapping to endpoint roles

The above five tasks differ in potential use as digital endpoints. The screening & risk stratification task is more suitable for population risk stratification and clinical trial recruitment, rather than as stand-alone diagnostic standards. The diagnosis & differential diagnosis task focuses on feasibility, accuracy, and consistency, and is  still far from formal diagnostic standards or consensus diagnostic indicators [175, 224]. The longitudinal monitoring task is more likely to serve as sensitive secondary or exploratory endpoints, or even primary endpoints, in drug and device clinical trials, particularly for capturing subtle symptom changes and continuous symptom fluctuations, and assessing real-world quality of life [171, 181, 190]. The prognosis & event prediction task is mostly retrospective or small-scale prospective. Translation into clinical tools with transparency, interpretability, and quantifiable risk–benefit, and integration into guidelines and regulatory frameworks, are important future directions [222]. Therefore, these different tasks should be taken into consideration in further study design and validation [225].

### Evaluation, validation, and regulatory considerations

PD digital biomarkers are typically evaluated in measurement performance (e.g., reliability, reproducibility, validity, and responsiveness), classification performance (e.g., accuracy, sensitivity, specificity, precision), generalization (cross-center and cross-device consistency) and fairness (e.g., equitable performance across regions).

#### Measurement properties & fit-for-purpose validation

At the measurement level, reliability is often assessed by repeating the same digital assessment in clinically stable patients over short intervals and quantifying agreement using tools such as intraclass correlation coefficients, correlation coefficients, or Bland–Altman analysis [165, 189]. Reproducibility emphasizes the consistency obtained by repeated measurements under identical conditions (including experimental protocol and devices) [226]. Validity is examined by relating digital indices to existing clinical definitions and external criteria, including associations with MDS-UPDRS scores, clinician-rated motor states, and patient-centered outcomes such as motor fluctuations, dyskinesia, or ON/OFF states [227]. Responsiveness means whether the measurements change appropriately with clinically meaningful interventions or disease progression [158, 228].

At the model-performance level, classification (e.g., PD versus controls, disease differential diagnosis, FoG occurrence) is typically evaluated using accuracy, sensitivity, specificity, precision–recall, F1 score, and area under the receiver operating characteristic curve. Corresponding reports can be found in studies on FoG detection and PD screening, diagnosis, and differential diagnosis [154, 167, 175, 229]. Regression tasks (e.g., severity prediction), such as mapping digital features to continuous clinical scales, are typically evaluated using standard regression metrics (e.g., *R*^2^ and mean absolute error) [164, 169].

#### Generalizability and fairness

Some studies have partially addressed fairness by reporting age and sex distributions across subtypes [167]. Cross-device consistency has been examined by comparing identical parameters measured by different devices [169]; and cross-center consistency and generalizability by comparing model performance across centers [230]. Future standard evaluation framework for digital biomarkers should systematically assess the fairness (sex, age, language/region), robustness and comparability of biomarker measurements across devices, and generalizability across regions, centers, and cohorts.

#### Alignment with the V3 framework

To align with commonly used validation terminology, the above evaluation layers can be mapped onto the V3 framework, which consists of (1) verification, addressing whether the sensors and systems measure the signals accurately; (2) analytical validation, assessing whether algorithms reliably derive measures; and (3) clinical validation, demonstrating associations with clinical anchors and meaningful outcomes.

#### Regulatory considerations

Future research on PD digital biomarkers should complement traditional measurement properties or AI model performance metrics with validation tiers aligned with regulatory and guideline requirements. Prospective validation across multinational, multicenter, and multi-ethnic populations is needed to support the development of digital endpoint measures that can be adopted in clinical trial, drug or device development programs, and regulatory decision-making [173, 225]. Emerging approaches such as synthetic data generation and privacy-preserving data augmentation may help address data scarcity and improve model robustness. However, additional validation is needed to confirm that the generated or augmented data are physiologically plausible and do not introduce artificial or biased patterns. Synthetic data should therefore be considered supportive evidence, not a substitute for prospective, real-world validation.

### Mechanistic interpretation

Mechanistic interpretability does not require digital biomarkers to directly measure specific neural circuits; rather, digital phenotypic changes should be interpretable within established pathophysiological frameworks.

Interpretability and disease mechanistic consistency are essential for incorporating digital biomarkers into a multidimensional pathophysiological characterization framework of PD. In previous work, clinically intuitive features of motor function, behavior, facial expression, and speech, were interpreted from the clinical and disease perspectives. For example, fusion of models based on articulation, phonation, and prosody can accurately distinguish PD from ET (and healthy controls). This is derived from the pathological relevance of speech motor control in PD and ET. Other studies reported that video-based hand-tracking kinematics (movement amplitude, tapping speed, rhythm stability, amplitude decrement) correlate with clinical bradykinesia ratings and can distinguish PD from controls. This aligns with the classic phenotypes of hand motor impairment in PD, suggesting the potential of video-based hand motor features as interpretable digital biomarkers [211, 224]. Overall, these strategies are beginning to link sensor-derived quantitative indices to potential disease mechanisms. In parallel, explainable AI techniques (e.g., feature-attribution methods such as SHAP [SHapley Additive exPlanations]) can help quantify contributions of inputs to model outputs, bridging algorithmic decisions and clinically interpretable phenotypes. Interpretability evidence that connects digital biomarker features with known disease mechanisms and clinically meaningful symptoms is particularly important when digital biomarkers are intended to support key clinical trial endpoints or treatment decisions. Together with traditional statistical measures and AI performance metrics, such evidence provides an important basis for evaluating the acceptability of digital biomarkers [231, 232].

### Integration with traditional biomarkers and future outlook

As a new-generation biomarker system, digital biomarkers have broader acquisition settings, higher temporal resolution, and higher sensitivity, enabling continuous, dynamic, and precise characterization of disease progression. In the context of PD, digital biomarkers can directly capture features of motor and behavioral abnormalities and thus offer advantages in treatment-response evaluation, capturing short-term disease-modification effects, and continuous monitoring of motor fluctuations [157, 182, 186, 228].

More importantly, digital biomarkers are complementary to traditional biomarkers. The digital measures capture external dynamic phenotypes of disease, whereas traditional biomarkers reflect internal structural, functional, and molecular pathology. Their integration can not only improve early identification, diagnostic characterization, therapeutic efficacy evaluation, and progression assessment, but also strengthen the basis for digital endpoints in drug/device trials and augment traditional clinical evidence with real-world evidence [233, 234].

## Summary and future directions

The landscape of PD biomarkers has evolved dramatically from clinical phenomenology towards a multi-modal, biology-driven framework. The convergence and integration of these biomarker domains hold the key to precision medicine in PD. Fluid/tissue biomarkers offer molecular and pathological specificity, imaging provides anatomical and functional localization within the brain, and digital biomarkers deliver continuous, real-world phenotypic quantification.

The αSyn-SAA using CSF or tissues (such as the skin) serves as the gold standard for confirming the ‘S + ’ status. Emerging αSyn PET tracers (such as [^18^F]C05-05) offer a promising proof-of-concept for visualizing intracerebral pathological burden in vivo. However, these tracers currently remain experimental. Future refinements to overcome existing technical barriers, such as off-target binding and strain-dependent sensitivity, are required before they can be used to support early diagnosis or precise disease staging in clinical settings. The imaging biomarkers alongside early digital motor function tests (such as frequency variations in fine finger movements), can be leveraged to characterize the degree of impairment in the ‘D’ dimension. Blood NfL concentration levels, the disappearance of the MRI swallowtail sign in the substantia nigra, and clinical motor impairment scores are utilized as measuring indicators for the ‘I’ dimension.

Future research must focus on longitudinal validation, standardization of assays and protocols (especially for αSyn-SAA, EVs, and digital feature extraction), and development of integrated multimodal algorithms. For clinical translation, future studies should also define the intended use of each biomarker, establish the evidence needed for its use in clinical trials or patient care, and determine whether digital measures can serve as reliable endpoints for monitoring disease progression or treatment response.

We here propose a preliminary tiered risk-stratification and case-finding funnel for precision medicine in PD. To be acknowledged, this framework is conceptual and requires prospective validation before broader implementation.

- Tier 1: Risk stratification and case-finding in enriched at-risk populations. Leveraging smartphone applications for speech analysis and movement analysis (including gait monitoring), this tier uses low-cost, non-invasive digital biomarkers to identify high-risk individuals within the enriched population. Algorithms are then employed to select those who require further examination.
- Tier 2: Clinic-feasible biological confirmation. In individuals with subjective motor concerns and/or quantifiable motor abnormalities on digital assessment from Tier 1, clinic-based peripheral testing may be considered to anchor synucleinopathy biology. This may include blood-based αSyn-SAA or skin αSyn testing. These minimally invasive approaches should not be used for population screening, nor should they be applied routinely to truly asymptomatic individuals. A positive result should be interpreted as evidence supportive of synucleinopathy biology (“S + ”) rather than as a stand-alone clinical diagnosis.
- Tier 3: Staging and subtyping. In a hospital setting, multimodal imaging (NM-MRI, DAT-SPECT) can help establish the “D + ” anchor and support staging within the NSD-ISS, while CSF or blood testing (e.g. NfL, Aβ/Tau) may further refine biological characterization and evaluate co-pathology.
- Tier 4: Monitoring. Medical-grade wearable sensors are used in home settings for longitudinal monitoring. During clinical trials, digital biomarkers enable high-frequency tracking of motor and non-motor impairments to capture subtle treatment-related changes over time.

A summary linking biological, imaging, and digital features with the NSD-ISS framework is provided in Table 6.

**Table 6:** A summary of multimodal features in relation to NSD-ISS staging

NSD-ISS stage	Biological features (Biofluids/Tissue)	Anatomical features (Imaging)	Functional (Digital Features)	Clinical implications
Stage 1 (Pre-Clinical)	αSyn-SAA positive (CSF/skin) Neurofilament light chain (NfL) normal	Structural MRI: normal Dopamine transporter (DAT) SPECT: possibly mild reduction	Subclinical digital signals: Altered keystroke dynamics Increased motor activity during sleep (RBD)	Optimal window for longitudinal observation and prevention-trial enrichment
Stage 2 (Prodromal)	SAA positive Inflammatory markers possibly elevated	Loss of nigrosome-1 (SWI) Reduced neuromelanin volume	Increased gait variability Reduced arm swing (passive monitoring) Subtle voice changes	Potential stage for prodromal enrichment and neuroprotection-oriented studies
Stage 3 (Clinical PD)	SAA positive NfL normal or mildly elevated	[^18^F]C05-05 signal in midbrain Significant striatal DAT loss	Quantified bradykinesia/tremor Detectable digital response to levodopa	Clinical stage in which symptomatic treatment optimization and biomarker-enabled monitoring may be relevant
Stage 4 + (Advanced)	NfL markedly elevated (neurodegeneration) Co-pathology markers (e.g., p-tau) present	Cortical atrophy Altered metabolic patterns on FDG-PET	Fall-risk alerts Freezing-of-gait (FoG) algorithm detection Monitoring of cognitive/speech impairment	Advanced stage in which complication monitoring and supportive care planning become increasingly important

This is a conceptual synthesis intended to illustrate how representative multimodal features may relate to the NSD-ISS. It is not based on prospective trial datasets, and it should not be interpreted as an established staging algorithm. *α-Syn-SAA* αSyn seed amplification assay, *CSF* Cerebrospinal fluid, *NfL* Neurofilament light chain, *MRI* Magnetic resonance imaging, *DAT* Dopamine transporter, *SPECT* Single-photon emission computed tomography, *RBD* REM sleep behavior disorder, *SWI* Susceptibility-weighted imaging, *FDG-PET* Fluorodeoxyglucose positron emission tomography, *p-tau* Phosphorylated tau

## Conclusion

Multimodal biomarkers are reshaping PD from a purely clinical syndrome into a biologically defined spectrum. Integration of fluid and tissue biomarkers, imaging, and digital phenotyping may help operationalize frameworks such as NSD-ISS and SynNeurGe. With further validation and standardization, this approach could support earlier diagnosis, biologically informed patient stratification, and more sensitive evaluation of disease progression and treatment response.

## Data Availability

No datasets were generated or analysed during the current study.
